# Genome-wide association analysis identifies a natural variation in basic helix-loop-helix transcription factor regulating ascorbate biosynthesis via D-mannose/L-galactose pathway in tomato

**DOI:** 10.1371/journal.pgen.1008149

**Published:** 2019-05-08

**Authors:** Jie Ye, Wangfang Li, Guo Ai, Changxing Li, Genzhong Liu, Weifang Chen, Bing Wang, Wenqian Wang, Yongen Lu, Junhong Zhang, Hanxia Li, Bo Ouyang, Hongyan Zhang, Zhangjun Fei, James J. Giovannoni, Zhibiao Ye, Yuyang Zhang

**Affiliations:** 1 The Key Laboratory of Horticultural Plant Biology, Ministry of Education, Huazhong Agricultural University, Wuhan, China; 2 Boyce Thompson Institute for Plant Research, Cornell University, Ithaca, New York, United States of America; 3 Robert W. Holley Center, US Department of Agriculture–Agricultural Research Service, Ithaca, New York, United States of America; University of Minnesota, UNITED STATES

## Abstract

Tomato (*Solanum lycopersicum*) is one of the highest-value vegetable crops worldwide. Understanding the genetic regulation of primary metabolite levels can inform efforts aimed toward improving the nutrition of commercial tomato cultivars, while maintaining key traits such as yield and stress tolerance. We identified 388 suggestive association loci (including 126 significant loci) for 92 metabolic traits including nutrition and flavor-related loci by genome-wide association study from 302 accessions in two different environments. Among them, an ascorbate quantitative trait locus *TFA9* (*T**OMATO* *F**RUIT* *A**SCORBATEON CHROMOSOME* *9*) co-localized with *SlbHLH59*, which promotes high ascorbate accumulation by directly binding to the promoter of structural genes involved in the D-mannose/L-galactose pathway. The causal mutation of *TFA9* is an 8-bp InDel, named InDel_8, located in the promoter region of *SlbHLH59* and spanned a 5’UTR Py-rich stretch motif affecting its expression. Phylogenetic analysis revealed that differentially expressed *SlbHLH59* alleles were selected during tomato domestication. Our results provide a dramatic illustration of how ascorbate biosynthesis can be regulated and was selected during the domestication of tomato. Furthermore, the findings provide novel genetic insights into natural variation of metabolites in tomato fruit, and will promote efficient utilization of metabolite traits in tomato improvement.

## Author summary

Deciphering the diverse, interconnected plant metabolome can facilitate crop improvement. In this study, the use of a combination of multiple technologies has allowed us to obtain novel functional and genetic insights into our GWAS investigating variation in ascorbate accumulation in tomato. The InDel_8 in the promoter of *SlbHLH59* was selected during tomato domestication and determines fruit ascorbate content by directly regulating the expression of structural genes involved in ascorbate biosynthesis in tomato fruit. The genes and polymorphisms responsible for the variations identified in this study lay the foundation for further comparative genomic studies and for improving nutrition quality in tomato and other fruit crops.

## Introduction

Tomato represents an important source of nutrients and fiber for the human diet and is a model system for studying fruit biology [1]. Cultivated tomatoes carry only a small fraction of the available genetic variation in this crop, since breeders have primarily focused on fruit size and stress resistance [2, 3], resulting in decreased flavour quality [4]. To address this issue, breeders must focus on quality as well as high yield [5]. Recent progress has been made in analysing the nutritional and flavour qualities of tomato, which can be assessed by assaying a range of metabolites whose selection can influence organoleptic and nutritional qualities [6, 7].

The diverse metabolites produced by plants can be divided into primary metabolites and secondary metabolites, in which primary metabolites play a central role in plant growth, cellular replenishment, resource allocation, and differentiation [8]. Primary metabolites include a wide range of intermediate compounds (such as sugars, organic acids, and amino acids) involved in glycolysis, the tricarboxylic acid (TCA) cycle, and amino acid metabolism [9]. Ascorbic acid (AsA) is an organic acid that scavenges reactive oxygen species and dietary AsA can reduce the incidence of important human diseases such as hypertension and diabetes [10]. Four pathways of ascorbate biosynthesis have been established in higher plants [11, 12], in which D-Man/L-Gal pathway, starting from glucose, is considered the most important in plants, and genes underlying all biosynthetic steps have been identified [13]. In this pathway, PMM mediates the interconversion between mannose 6-phosphate and mannose 1-phosphate, and is required for the synthesis AsA in both Arabidopsis and *N*. *benthamiana* [14]. GMP, a rate-limiting enzyme of the D-Man/L-Gal pathway, catalyzes the conversion of D-mannose-1-P to GDP-D-mannose [15]. An ozone-sensitive Arabidopsis mutant showing significant reduction of AsA has been mapped to the VTC1 locus encoding a GDP-D-mannose pyrophosphorylase [16]. The mRNA levels of GMP are correlated with L-ascorbate levels in several plant species [17, 18].

An additional important quantitative trait for the tomato processing industry is fruit soluble solids content (SSC), which primarily reflects a combination of fructose, glucose and additional sugars. Altering the content and proportion of sugars and acids is a major breeding strategy for improving the flavour of processing tomato [19]. Several QTLs has been identified in tomato which influence these traits, including *Lin5* and *SSC11*.*1* [20]. Given their important role, understanding the genetic basis of variations in nutrition and flavor related metabolites among diverse tomato varieties will provide important insight for efforts to facilitate breeding of elite varieties with enhanced nutritional content and improved flavour.

Metabolomic quantitative trait locus (mQTL) mapping in bi-parental populations is an effective method for exploring the genetic architecture of primary metabolites. Valuable QTLs specific to the parental lines of mapping populations have been detected, including several candidate causal genes [21], and major genes involved in regulating primary metabolism in tomato [2, 3], but our understanding of natural variation in primary metabolites in natural populations and in a given plant species remains limited. Technological developments have extended our ability to understand the genomic diversity in tomato, facilitating the analysis of metabolites and locus–locus interactions in plants [22–24]. Genome-wide association studies (GWAS) of metabolic traits enable screening of numerous accessions to explore the genetic basis of metabolic diversity. For example, the high-quality reference genome and rich re-sequencing data have facilitated GWAS investigations of primary metabolic traits in tomato [23, 25–28]. Using a large population and meticulous genotyping, Tieman *et al*. (2017) drew a genetic roadmap related to tomato flavour to facilitate the breeding of high-quality modern commercial varieties [20].

In the present study, we performed GWAS for 92 metabolic traits using 302 diverse tomato accessions that were characterized in two different years and environments and genotyped with 4,180,023 SNPs. We uncovered a relatively simple genetic architecture for most metabolic traits. A novel transcription factor (TF) *SlbHLH59* for ascorbate underlying one of these QTLs was functionally and phylogenetically characterized suggesting *TFA9* was a domestication target. These findings provide new information on the genetics of fruit quality and provide a foundation for additional discovery of the genetic regulation of metabolic traits.

## Results

### Metabolite profiling and its genetic basis in tomato

By combined metabolomic approach (see Method), we detected and quantified 92 metabolic traits in red fruit from the association panel (302 inbred lines) harvested in two environments open field (E1) and greenhouse (E2) (S1–S3 Tables). Most metabolites had coefficients of variation (*CV*) was greater than 40% (S1A Fig). Among the 49 repeatedly detected traits (48 primary metabolites including ascorbate, SSC), 46.9% (23 out of 49) displayed broad-sense heritability (*H*^*2*^) greater than 0.3, and 22.4% had heritability values >0.5 (S1B Fig). A wide range of variation was observed for some metabolites in each species and subgroup over the years (S4 Table). Across the groups, several sugars (sucrose, fructose, and *myo*-inositol) and organic acids (citric acid, malic acid, ascorbic acid and pentanoic acid) were present at the highest levels in PIM, as were two amino acids (alanine and L-glutamic acid) and three flavor-related components (SSC, total sugar and total acid) (S2 Fig), suggesting selection during domestication for these metabolites.

**Fig 1:**
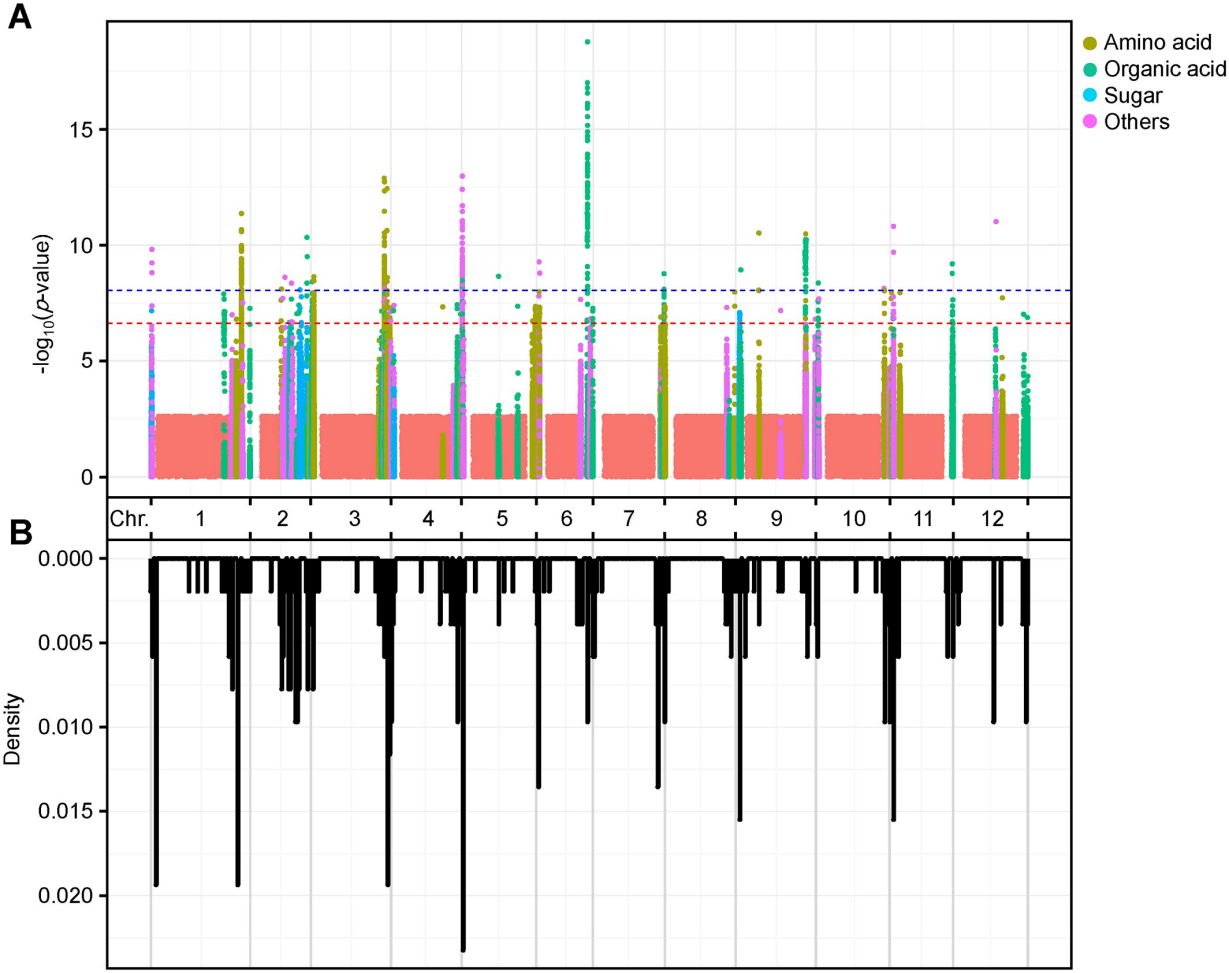
Overview of mGWAS results in this study. **(A)** The strength of the association of metabolites is indicated as the negative logarithm of the *P* value for the CMLM model. All metabolite–SNP associations with *P* values < 2.4 × 10^−7^ are plotted against genome location at 1-Mb intervals. Red and blue horizontal dashed lines in the manhattan plots indicate the genome-wide suggestive (*P* values < 2.4 × 10^−7^) and significant (*P* values < 1.2 × 10^−8^) threshold, respectively. Amino acids are indicated by olive green circles. Organic acids are indicated by light green circles. Sugars are indicated by light blue circles. Other compounds are indicated by pink circles. **(B)** Distribution density of mGWAS signals across the tomato genome (SL2.50). mGWAS, metabolomic genome wide association analyses.

We performed GWAS using a compressed mixed linear model (CMLM) to reveal the metabolic regulatory mechanisms in tomato fruit under different environments (E1 and E2). Using a Bonferroni correction based on the effective numbers of independent markers [29], the *P*-value thresholds were set at 2.4 × 10^−7^ (suggestive) and 1.2 × 10^−8^ (significant). The detected 388 lead SNPs (including 262 suggestive SNPs and 126 significant SNPs) across the two environments (S3 Fig and S5 Table), including repeated detection of 103 SNPs (Fig 1). We identified six potential mGWAS hotspots (density>0.015), on chromosomes 1, 3, 5, 9, and 11. These loci were often frequently by metabolites that are biochemically related; for instance, a hot spot on chromosome 1 was identified for approximately half (46.7% or 7/15) of the sugars detected in this study (S5 Table). Candidate genes underlying these loci might encode central regulators of these pathways and/or influence rate-limiting reactions. The percentage of phenotypic variation explained by each locus ranged from 2.4 to 36.1% and from 2.1 to 30.3% in the two replicates, with mean values of 10.2 and 8.6%, respectively (Table 1). To test for possible interactions between high significance QTL loci (*P*≤1.2 × 10^−8^), we investigated the pairwise epistatic interactions between the QTLs of each metabolic trait in each environment. We detected 27 significant interactions (*P*<0.05) for 4 metabolites (4-hydroxyproline, quininic acid, lactic acid and galactose oxime) whose levels were controlled by more than one QTL in both environments (S6 Table). The epistatic effect (i.e., sum of two-locus interaction effects) on metabolic variation ranged from 2.2 to 51.2%, with an average of 8.03%, suggesting that epistasis plays an important role for the four metabolites. For lactic acid, the epistatic effect was weaker compared with the main effects of the loci (i.e., sum of single-locus effects), while for the other three metabolites, the effect was comparable to or greater than the predominant effect (S4 Fig) suggesting diverse pathways or interconnected mechanism.

**Table 1:** Summary of significant loci–trait associations identified in GWAS population.

Item	E1^a^	E2	Detected in at least one population	Detected in both population
Number of metabolic traits^b^	58/66	55/74	92	49
Number of Suggestive SNPs (Significant SNPs)^c^	245(73)	243(91)	388(126)	103(38)
Number of loci per trait (Range)^d^	1–16	1–14	1–19	1–7
SNPs above 15% of variation	37	45	50	33
Maximum explained variation (%)	36.1	30.3	36.1	36.1
Explained variation per SNP (%)	10.2	8.6	8.5	13.1

^a^E1 and E2 represent the two experiments conducted on the association panel.

^b^Number of traits having significantly associated loci or QTL (before slash), number of total detected traits (after slash).

^**c**^Number of association loci detected in each experiment on the association panel (Suggestive SNPs, *P*≤2.4×10^−7^; Significant SNPs, *P*≤1.2×10^−8^). Lead SNPs means the SNPs with the lowest *P* value in a defined region.

^d^Number of significant loci detected per trait.

### Key genes involved in primary metabolism in ripe tomato fruit

The high-density linkage disequilibrium map generated in this study helped us narrow down association signals to regions close to or directly on genes that have been reported previously [3, 27, 30–35]. For each significant loci identified in this study, candidate genes within 200kb (<average LD of tomato) of the lead SNP are listed in S5 Table, providing a database for investigation of specific metabolites of interest.

Taking advantage of the mGWAS results, we searched for candidate genes based on (i) gene annotation, (ii) prior knowledge, (iii) gene expression, and (iv) structural variation to identify the most likely causal genes of the identified loci for metabolites measured here. The 37 candidate genes listed in S7 Table, which are located within 32 significant loci for 27 metabolites, are potentially causative for the identified association signals (S5 Fig). Among these 37 candidate genes, four significantly associated loci (ch02_44249907, ch05_1882379, ch06_36888571, and ch09_3306649) on chromosomes 2, 5, 6, and 9, with *P* values of 1.4 × 10^−7^, 2.8× 10^−8^, 3.4 × 10^−9^ and 3.9 × 10^−11^ were identified by GWAS for SSC. Three of these four loci correspond to *HT1* (Hexose transporter 1), *SUT2* (Sucrose transporter LeSUT2), and *Lin5* have been reported previously [3, 31, 32]. Ch06_36888571 is a novel locus within LD of *Solyc06g054270*, which encodes the sugar transporter STP11. We further validated the association between its allelic variations and SSC via mQTL mapping (S6 Fig**)**. We generated an experimental F_2_ population by crossing a tomato accession with low SSC, HG22 (an elite inbred line in China, relative SSC of 4.8%) with TS-21, which confers high SSC (a *Solanum pimpinellifolium* accession from Peru, relative SSC of 9%). By the bulk segregant analysis (BSA), the causal locus of fruit SSC accumulation was mapped to two intervals, 2.07 Mb on Chr 2 and 7.84 Mb on Chr6 (from ~38.8 to 40.8 Mb on Chr2 and 36.1 to 43.9 Mb on Chr6, respectively), with the peak centered on the mapping interval identified in our GWAS analysis (S6A and 6H Fig). The results of linkage mapping in the F_2_ population from TS-21×HG22 were consistent with the results of GWAS analysis, further supporting the notion that *STP11* on chromosome 6 is a candidate gene driving the observed natural variation in fruit SSC (S8 Table). This demonstrates that our metabolite profiling and GWAS analysis could provide accurate genetic architecture of tomato primary metabolites. We further investigate the putative loci associated with ascorbate concentration in tomato fruit. Three loci (SL2.50ch07_1311726 with *P* value = 8.0 × 10^−8^, SL2.50ch07_60983724 with *P* value = 4.6 × 10^−10^ and SL2.50ch07_65942036 with *P* value = 4.1 × 10^−11^) on chromosome 7 and one loci (ch09_64101874 with *P* value = 3.1 × 10^−11^) on chromosome 9 were identified by the GWAS of AsA.

### Identification of *TFA9* by GWAS

**Fig 2:**
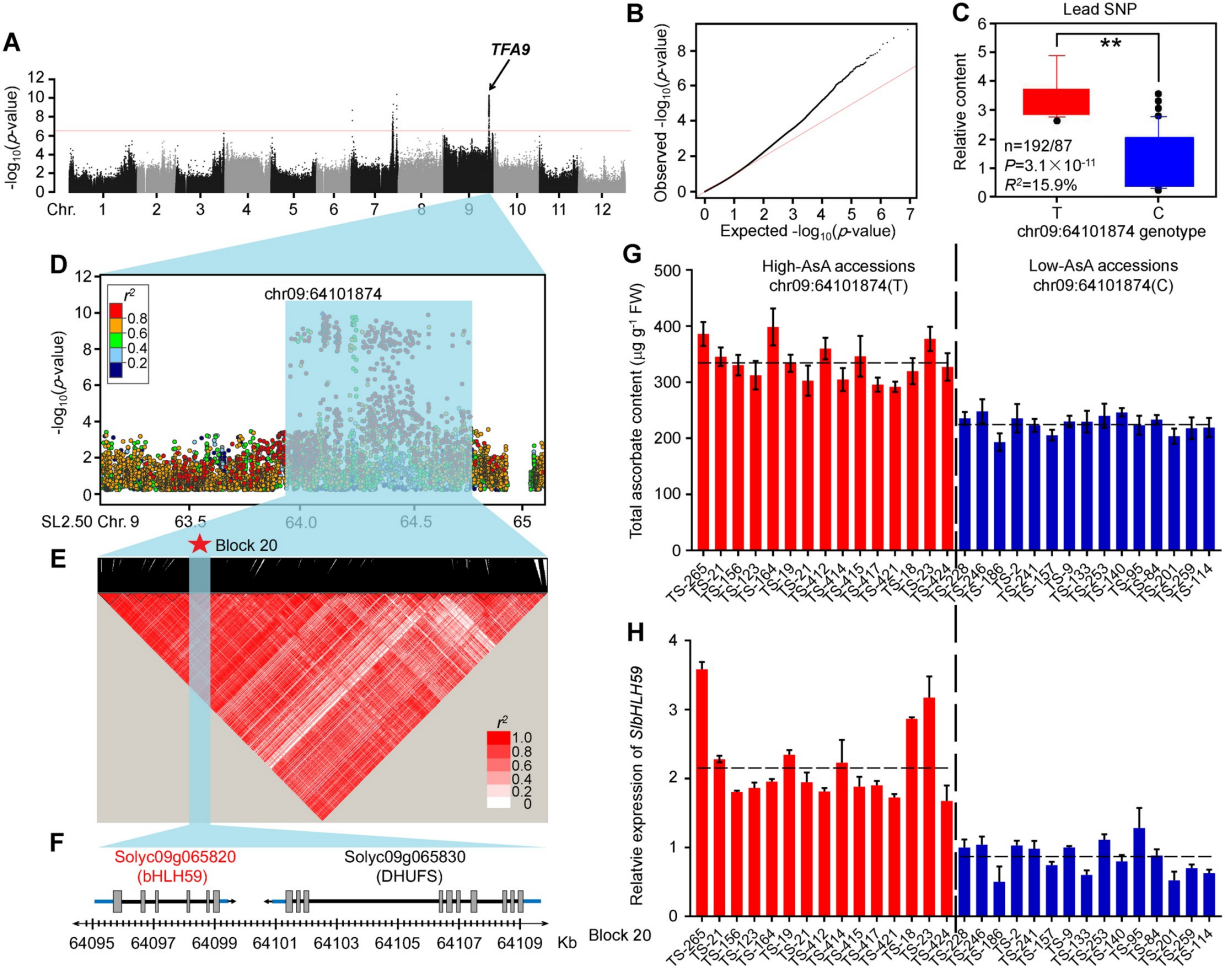
Genome-wide association results for ascorbate content in tomato fruits. **(A)** Manhattan plot displaying the GWAS results for ascorbate content in fruits (CMLM, *N* = 302). Negative log_10_-transformed *P* values from the compressed mixed linear model are plotted on the *y-*axis. Horizontal dashed line indicates a genome-wide suggestive threshold of 2.4×10^−7^. **(B)** Quantile-quantile plot for ascorbate content in the GWAS population. **(C)** Box plot for ascorbate content, plotted as a function of genotypes at SNP ch09_64101874. The metabolic data for ascorbate were log_2_ transformed. **(D)** Detailed plot from 63.1–65.1 Mb on chromosome 9 (*x-*axis). Lead SNP is indicated in purple. A representation of pairwise *r*^*2*^ values (a measure of LD) among all SNPs in region 63.1–65.1 Mb, where the colour of each box corresponds to the *r*^*2*^ value according to the legend. **(E)** A representation of the pairwise *r*^2^ values (a measure of LD) among all polymorphic sites in the 830 kb genomic region corresponding to **(D)**, where the darkness of the color of each box corresponds to the *r*^2^ value according to the legend. The 145 haploblocks are represented by inverted triangle. Haploblock 20 (marked by red star) contains Lead SNP associated with fruit AsA content. **(F)** Gene structure of two genes in the haploblock 20. Filled grey, blue and blacklines represent coding sequence, promoter & 3’UTR and introns respectively. **(G, H)** The ascorbate content **(G)** and relative expression of candidate gene (*SlbHLH59*) **(H)** in fruits from different selected accessions. Data represent means ±s.d. (n = 3). The dashed horizontal lines represent the average ascorbate content and expression levels of *SlbHLH59* in fifteen low-AsA accessions and fifteen high-AsA accessions.

Among those loci associated with ascorbate concentration in tomato fruit, one loci was co-localized with a previously reported AsA large-effect QTL on chromosome 9 [36], we designated this locus as *TOMATO FRUIT ASCORBATE ON CHROMOSOME 9* (*TFA9*) **(**Fig 2**)**. The SNP with the highest association to fruit AsA content explained 15.9% of the total variance. Two major haplotypes based on the lead SNP (ch09_64101874) of the association signal—High-AsA haplotype (HAH) and Low-AsA haplotype (LAH)—were associated with high-AsA and low-AsA phenotypes in tomatoes, respectively **(**Fig 2C**)**.

There was a total of 19 genes within 100-kb on either side of ch09_64101874 (S9 Table). But given the estimated LD decay rate of more than 800 kb in BIG group tomato [26], we carefully analyzed the pairwise LD distance within the 2-Mb interval centered on the lead SNP (ch09_64101874) from the GWAS **(**Fig 2D**)**. All significant SNPs (*P* value ≤ 1.2 × 10^−8^) fall into an 830 Kb region for 63.95 Mb to 64.77. A haplotype analysis of the region spanning all the significant SNPs on chromosome 9 (830 Kb) identified 145 haploblocks, and many significant SNPs including lead SNP (ch09_64101874) trace back to haploblock 20 (SL2.50ch09_64,095,308-SL2.50ch09_64,109,883) (Fig 2E). The Haploblock 20 (14.575 kb; Fig 2F) spans two genes, a bHLH transcription factor (*Solyc09g065820*, 64,095,481–64,100,029bp) and NADH ubiquinone oxidoreductase (*Solyc09g065830*, 64,101,025–64,109,232bp) and contains 47 SNPs, 19 of which show *P* values were less than 12.2× 10^−7^ (S10 Table), suggesting their potential role in AsA accumulation. To identify the casual gene for AsA content in tomato fruit, we randomly selected 15 PIM accessions of high-AsA and 15 BIG accessions of low-AsA and measured expression of both genes in fruit by quantitative RT-PCR. The expression of *Solyc09g065820* showed significantly higher expression in fruit with high-AsA as compared to low-AsA accessions (Fig 2G and 2H). No significant difference was observed in the expression of NADH ubiquinone oxidoreductase (S7 Fig). Basic helix-loop-helix (bHLH) proteins are a large superfamily of transcription factors functioning in a wide range of metabolic, physiological, and developmental processes in plants [37, 38]. Based on these results, *Solyc09g065820* gene (referred to hereafter as *SlbHLH59*) is the likely candidate underlying *TFA9*.

### An 8-bp InDel in the promoter of *SlbHLH59* influences its expression

**Fig 3:**
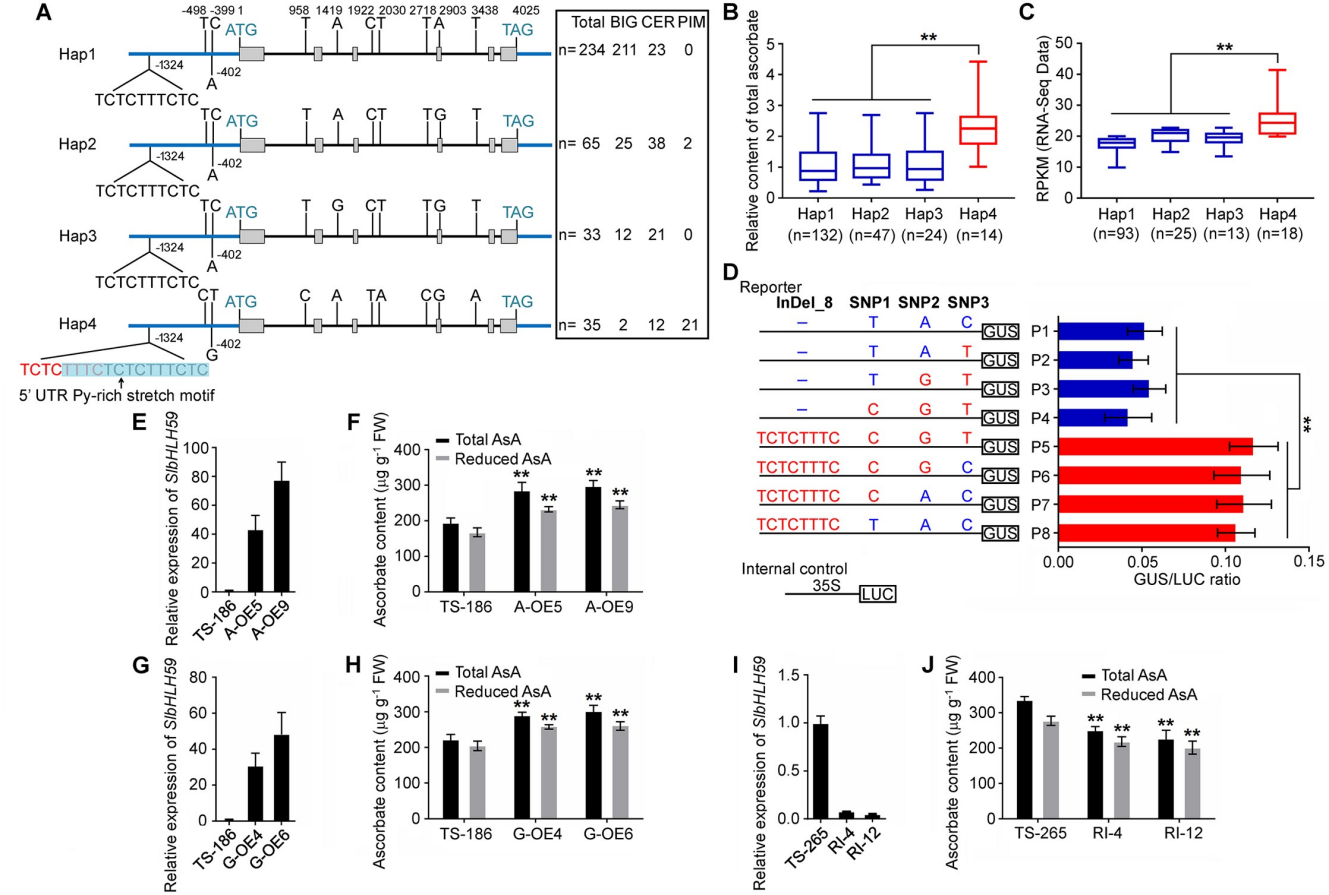
Haplotype and functional analysis of *SlbHLH59* in tomato. **(A)** Structural variations of the four *SlbHLH59* haplotypes. Blue represent the promoter and 3’ UTRs, grey boxes represent coding sequences and the line between the grey boxes represents the intron. The 11 nucleotide polymorphisms are indicated at their corresponding positions. Four haplotypes of *SlbHLH59* are examined in 367 tomato accessions. **(B, C)** The relative ascorbate content **(B)** and expression of *SlbHLH59* **(C)** in fruits of four *SlbHLH59* haplotypes. **(D**) Transient expression assay of promoter activity in *Arabidopsis* protoplasts. The *SlbHLH59* promoter fragments were inserted into the reporter vector (pCAMBIA1304-GUS). Both vectors of reporters and internal control (pGreen II 0800, 35S+LUC) were transformed into *Agrobacterium* cells and used for *Arabidopsis* protoplast transformation. Left, constructs with site-directed mutations at the four polymorphisms in the promoter region. Right, relative GUS/LUC values. Values are represented as means ± SD (n = 3). **(E, G, I)** The relative expression of *SlbHLH59* in *SlbHLH59*^*SNP9A*^ overexpression **(E)** *SlbHLH59*^*SNP9G*^ overexpression **(G)** and *SlbHLH59* RNAi **(I)** transgenic tomato lines. **(F, H, J)** The ascorbate content in *SlbHLH59*^*SNP9A*^ **(F)** *SlbHLH59*^*SNP9G*^ **(H)** and *SlbHLH59* RNAi **(J)** transgenic tomato lines. The background of *SlbHLH59* overexpression and RNAi transformation is TS-186 and TS-265 respectively. Asterisks indicate significant differences by *t* test: **P< 0.01.

To investigate functional allelic variation at the *SlbHLH59* locus, we analyzed the nucleotide sequence of *SlbHLH59* in 369 tomato accessions with diverse AsA content. Sequence analysis suggested that the *SlbHLH59* genotype can be classified into four different haplotypes (Hap 1, Hap 2, Hap 3 and Hap 4) by a total of 11 polymorphisms (Fig 3A and S11 Table), including one InDel (InDel_8, ->TCTCTTTC variant at position -1324) and three SNPs (SNP1, T>C variant at position -983; SNP2, A>G variant at position -402; SNP3, C>T variant at position -399) in the promoter region, six intron SNPs (SNP4, T>C variant at position 958; SNP5, A>G variant at position 1419; SNP6, C>T variant at position 1922; SNP7, T>A variant at position 2030; SNP8, T>C variant at position 2718; SNP10, T>A variant at position 3438), and one nonsynonymous polymorphism in exon 4 (SNP9, A>G variant at position 2903, with amino acid change from I to V). Interestingly, Hap 1 (mainly consists of BIG accessions), Hap 2 (consisting of BIG and CER accessions) and Hap 3 (consisting of BIG and CER accessions) showed lower AsA content than Hap 4 (mostly PIM accessions) (Fig 3B), and line with the result mentioned above where PIM tomato accessions showed higher AsA content than CER and BIG tomato accessions **(**S2B Fig**)**.

All accessions in Hap 4 with nine consensus polymorphisms (InDel_8, SNP1, SNP2 and SNP3 in promoter; SNP4, SNP6, SNP7, SNP8 and SNP10 in intron) exhibited higher AsA content than other haplotypes suggesting variation in AsA content among the *SlbHLH59* haplotypes was attributed to polymorphisms in the promoter (Fig 3A). The two SNPs (SNP1 and SNP2) do not change the known *cis* elements in the promoter of *SlbHLH59* but SNP3 resides at a box-1 *cis* element (light responsive element) according to PLACE (Plant cis-acting regulatory DNA elements) analysis. Notably, the InDel_8 in the promoter of *SlbHLH59* led to the formation of a 5’UTR Py-rich stretch motif (TTTCTCTCTTTCTC) associated with elevated expression of downstream genes [39, 40], and consistent with increased *SlbHLH59* expression in the high-AsA accessions that contain this motif versus the low-AsA accessions without this motif **(**Fig 2H**)**. Using published data [41], we observed that *SlbHLH59* showed significantly higher expression in Hap 4 accessions than in other haplotypes (Fig 3C). Moreover, we conducted transient assays using site-mutated promoter fragments of *SlbHLH59* in *Nicotiana benthamiana* to test the effects of the four polymorphisms (three SNPs and InDel _8) under the promoter region on *SlbHLH59* expression **(**Fig 3D**)**. Expression was significantly higher in the promoter fragments with the 8bp insertion than not, supporting its role in the expression of *SlbHLH59*.

In total of 159 tomato bHLH proteins, 68 SlbHLHs (43%) showed I and 60 SlbHLHs (38%) showed V at the SNP9 which is located on the second helix of the conserved bHLH domain, suggesting the conservation of this amino acid residues in tomato **(**S8A Fig**)** [42]. The non-consensus SNP9 with “A” allele in Hap 1 and “G” allele in Hap 2, 3 and 4 showed irrelevance with AsA content in tomato (Fig 3A). To further functionally characterize the role of SNP9on *SlbHLH59* expression and AsA content, two overexpression (OE) constructs containing allele *SlbHLH59*^*SNP9A*^ and *SlbHLH59*^*SNP9G*^ were separately introduced into TS186 (a low AsA accession). The two *SlbHLH59*^*SNP9A*^ transgenic plants, A-OE5 and A-OE9, with higher *SlbHLH59* expression, showed significantly enhanced total and reduced AsA content compared to wild type (Fig 3E and 3F). Similarly, fruits from G-OE4 and G-OE6, two *SlbHLH59*^*SNP9G*^ overexpression transgenic lines exhibited enhanced AsA content than the control, with the comparable AsA levels as A-OE5 and A-OE9 fruits **(**Fig 3G and 3H**)**. These results indicate that the SNP9A allele and SNP9G allele of *SlbHLH59* are both functional, consistent with the results of haplotype analysis indicating SNP9 was non-causal for AsA content (Fig 3A). The differential AsA accumulation within tomato is therefore more likely attributed to nucleotide differences in the promoter region. To further test the function of *SlbHLH59* in AsA biosynthesis, an RNA interference (RNAi) vector was constructed and transformed into TS-265 (a high AsA accession). Down regulation of *SlbHLH59* in TS-265 resulted in significant reduction AsA content **(**Fig 3I and 3J**)**. All of these results suggested that the InDel_8 in the promoter of *SlbHLH59* and the resulting absent/present 5’UTR Py-rich stretch motif, is the major cause underlying the QTL *TFA9* on variation in AsA levels and attributable to altered gene expression.

### Expression pattern of *SlbHLH59*

**Fig 4:**
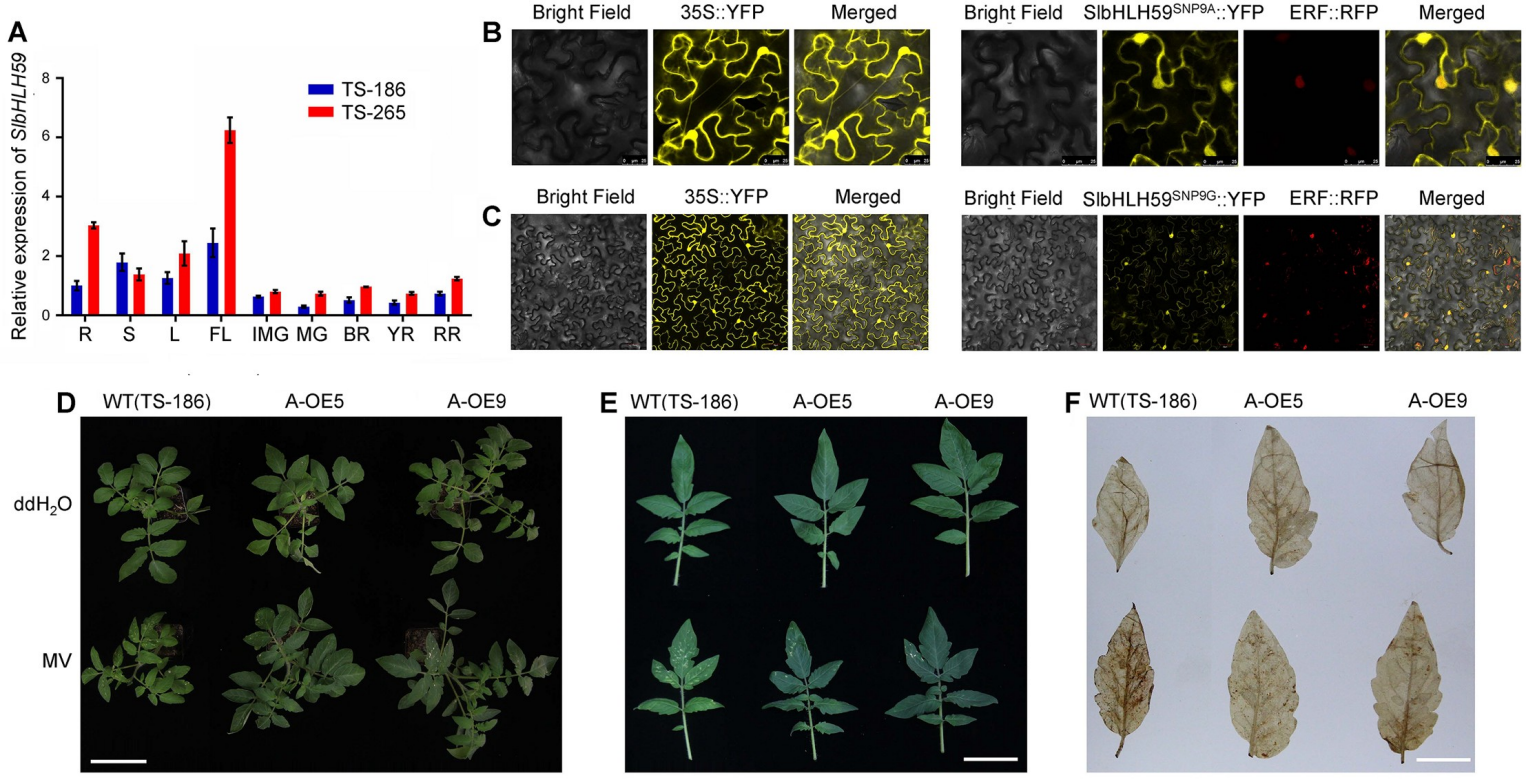
The characterization of *SlbHLH59*. **(A)** The transcript levels of *SlbHLH59* indifferent tomato organs: R, root; S, stem; L, leaf; FL, flower; IMG, immature fruit; MG, mature green fruit; BR, breaker stage fruit; YR, yellow stage fruit; RR, red ripe stage fruit. TS-186 is a low-AsA accession; and TS-265 is a high-AsA accession. **(B, C)** Subcellular co-localization of transiently expressed SlbHLH59^SNP9A^-YFP **(B)** and SlbHLH59^SNP9G^-YFP **(C)** fusion protein with a nuclear marker (ERF) in *N*. *benthamiana* leaves. Bars = 50 μm. **(D-F)** Phenotype comparisons of *SlbHLH59*-OE and wild-type plants after methyl viologen (MV) treatment. The *SlbHLH59*-OE transgenic plants had less yellow lesion **(D, E)** and H_2_O_2_ accumulation **(F)** than WT after MV treatment. Bar = 10 cm **(D)**, 5cm **(E)** and 2cm **(F)**.

Phylogenetic analysis showed that SlbHLH59 belongs to the basic helix-loop-helix (bHLH) family transcription factors and showed highest amino acid similarity with UNE12 (UNFERTILIZED EMBRYO SAC 12) which is responsible for the regulation of fertilizationin processes in *Arabidopsis* [43] (S8B Fig and S1 File). We investigated the spatial and temporal expression patterns of *SlbHLH59* in high and low AsA content accessions (high-AsA accession TS-265 and low-AsA accession TS-186). *SlbHLH59* showed high expression in leaves but low in different fruit developmental stages, with the transcript level of *SlbHLH59* higher in most tissues of TS-265 versus TS-186 **(**Fig 4A**),** supporting a role of *SlbHLH59* in positively regulating AsA content in tomato. A previous study revealed that light plays a critical role in regulating AsA metabolism and accumulation [44]. To assess whether *SlbHLH59* is involved in light-dependent ascorbate metabolism, we analyzed the expression of *SlbHLH59* under successive illumination circulation. Interestingly, we observed light-suppressed *SlbHLH59* expression, i.e. it rapidly decreased in the light and increased under dark (S9 Fig). These results explained in part the molecular mechanism of light-dependent accumulation of AsA in tomato.

In order to investigate SlbHLH59 cellular localization, we created SlbHLH59^SNP9A^-YFP and SlbHLH59^SNP9G^-YFP fusion proteins, which were transiently expressed in *Nicotiana benthamiana*. Fluorescent signals of YFP overlapped with that of ERF-RFP, a marker for the nucleus, suggesting that both SlbHLH59^SNP9A^ and SlbHLH59^SNP9G^ were located in the cell nucleus (Fig 4B and 4C).

When plants were exposed to oxidative stress, AsA acts as an antioxidant protecting cells from oxidative damage by scavenging excessive reactive oxygen species [10]. To evaluate whether overexpressed *SlbHLH59* in tomato can increase tolerance to oxidative stress, 1-month-old A-OE lines (A-OE5 and A-OE9) and wild type (TS-186) were subjected to oxidative stress by treatment with 75 μM methyl viologen (MV) for 2 days. DAB staining showed that there was no significant difference between TS-186 and A-OE lines under normal conditions (treatment with ddH_2_O), but more brown spots in the leaves of TS-186 were observed than in the leaves of A-OE after MV treatment **(**Fig 4D–4F**)**. Additionally, the content of chlorophyll and MDA were not significantly altered in the A-OE lines, but significantly decreased and increased in the wild-type after MV treatment, respectively (S10 Fig). These results demonstrate that *SlbHLH59* induces AsA accumulation facilitating increased oxidative stress tolerance.

### *SlbHLH59* regulates AsA biosynthesis by directly modulating the expression of *SlPMM*, *SlGMP2* and *SlGMP3*

**Fig 5:**
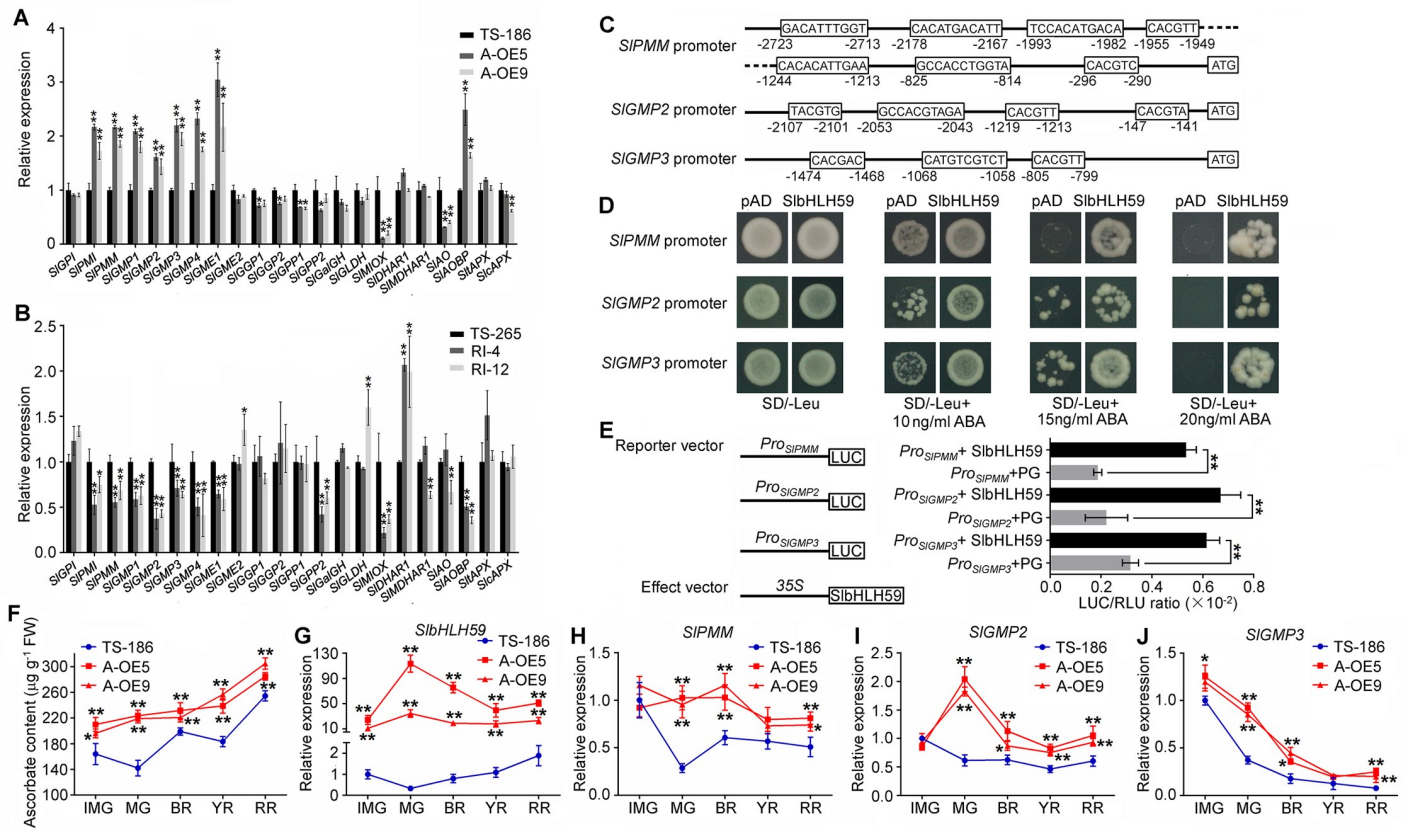
Identification of *SlPMM*, *SlGMP2* and *SlGMP3* as direct targets of SlbHLH59. **(A, B)** Relative expression of ascorbate (AsA) biosynthesis, recycling and oxidation-related genes in fruits of *SlbHLH59* overexpression **(A)** and RNAi **(B)** lines. **(C)** The promoter *cis*-elements of *SlPMM*, *SlGMP2* and *SlGMP3*. The sequences in black boxes represent E-box *cis*-elements. **(D)** Yeast-one hybrid (Y1H) assay of SlbHLH59 binding to promoter fragments of *SlPMM*, *SlGMP2* and *SlGMP3*. The bait vectors *SlPMM*_*pro*_, *SlGMP2*_*pro*_ and *SlGMP3*_*pro*_ (the fragments containing the region 3000 bpupstream from initiation codon) and the prey vector containing *SlbHLH59* were introduced into yeast strain Y187, and interaction between bait and prey enhanced ABA resistance. Yeast cells spread on SD-Leu media with various concentrations of ABA (0, 10, 15, and 20 mM). The bait vector (*SlPMM*_*pro*_, *SlGMP2*_*pro*_ and *SlGMP3*_*pro*_) + pGADT7 were also transformed into Y187 as a negative control. **(E)** Binding of SlbHLH59 to promoters of *SlPMM*, *SlGMP2* and *SlGMP3* assayed by dual luciferase system. The *SlbHLH59* ORF was cloned into the effector vector (pGreen II62-SK) and promoter fragments of *SlPMM*, *SlGMP2* and *SlGMP3* were inserted into the reporter vector (pGreen II 0800 LUC). Both vectors of effectors and reporters were transformed into *Agrobacterium* cells and used to infiltrate tobacco leaves. LUC, firefly luciferase activity; RLU, Renilla luciferase activity; PG, the empty vector of pGreenII 62-SK. The promoters of *SlPMM*, *SlGMP2* and *SlGMP3* plus PG were used as control. **(F-J)** Dynamics of ascorbate content and *SlbHLH59* expression during tomato fruit development. Ascorbate (AsA) concentration **(F)** and expression of *SlbHLH59* **(G)**, *SlPMM* **(H)**, *SlGMP2* **(I)**, *SlGMP3* **(J)** at different fruit developmental stages of wild-type (TS-186) and *SlbHLH59* transgenic lines. Experiments were performed in immature (IMG), mature green (MG), breaker (BR), yellow ripe (YR) and red ripe (RR) fruit, respectively, with three replicates. All data in the graphs are presented as means ± SE. Asterisks indicate significant differences by *t* test: *P < 0.05; ** *P*< 0.01.

bHLH transcription factors have previously been reported to recognize and bind to the E-box *cis*-element (CANNTG), thereby affecting the expression of downstream genes [45]. To test whether the expression of structural genes in the AsA biosynthesis pathway were modulated in the *SlbHLH59* transgenic plants, we performed qPCR analysis. The expression of *SlPMI*, *SlPMM*, *SlGMP1*, *SlGMP2*, *SlGMP3*, *SlGMP4* and *SlGME1* were higher and lower in the fruits of the *SlbHLH59*-OE and *SlbHLH59*-RI lines, respectively, when compared with wild-type fruits, suggesting transcriptional regulation mediated by *SlbHLH59* on the AsA biosynthetic genes **(**Fig 5A and 5B**)**. Using PLACE (http://www.dna.affrc.go.jp/PLACE/signalscan.html) and PlantCARE (http://bioinformatics.psb.ugent.be/webtools/plantcare/html/) software, we analyzed the *cis*-elements in the promoters of *SlPMI*, *SlPMM*, *SlGMP1*, *SlGMP2*, *SlGMP3*, *SlGMP4* and *SlGME1*, and determined that all of the genes were predicted to harbor the E-box *cis*-elements (S12 Table). We speculated that SlbHLH59 might bind and regulate these genes of the D-Man/L-Gal pathway. To test this hypothesis, we conducted yeast one-hybrid (Y1H) analysis to test the binding activity of SlbHLH59 protein to the promoters of *SlPMI*, *SlPMM*, *SlGMP1*, *SlGMP2*, *SlGMP3*, *SlGMP4* and *SlGME1*. *Cis*-elements from the *SlPMM*, *SlGMP2* and *SlGMP3* promoters were bound by SlbHLH59 **(**Fig 5C and 5D**)**. Also, the physical interactions between SlbHLH59 and the promoter fragments derived from *SlPMM*, *SlGMP2* and *SlGMP3* were detected by using the dual luciferase system **(**Fig 5E**)**. These results indicate that SlbHLH59 can directly bind to the *SlPMM*, *SlGMP2* and *SlGMP3* promoter to modify their expression, and thereby positively regulate tomato fruit AsA content.

To test how SlbHLH59 affects *SlPMM*, *SlGMP2* and *SlGMP3* during the fruit development in tomato, we determined total AsA and the expression of *SlPMM*, *SlGMP2* and *SlGMP3* in immature green stage (IMG), green mature stage (MG), breaker stage (BR), yellow ripe stage (YR) and red ripe stage (RR) fruits. The G-OE4 showed higher total AsA content than TS-186 throughout the whole fruit development stages and the greatest difference was observed at MG stage, consistent with the dynamic change of *SlbHLH59* transcript level **(**Fig 5F and 5G**)**. The expression of *SlPMM*, *SlGMP2* and *SlGMP3* were all significantly higher in G-OE4 than in TS-186 except *SlPMM* and *SlGMP2* in IMG fruits, and the greatest difference was observed at MG stage, consistent with the dynamic change of AsA content in TS-186 and *SlbHLH59* overexpression line **(**Fig 5H–5J**)**. This result supports the notion that *SlbHLH59* contributes to AsA biosynthesis by directly regulating *SlPMM*, *SlGMP2* and *SlGMP3* during fruit development with the highest effect at the MG stage just prior to the onset of ripening.

### Human selection of SlbHLH59^InDel_8^ in tomato

**Fig 6:**
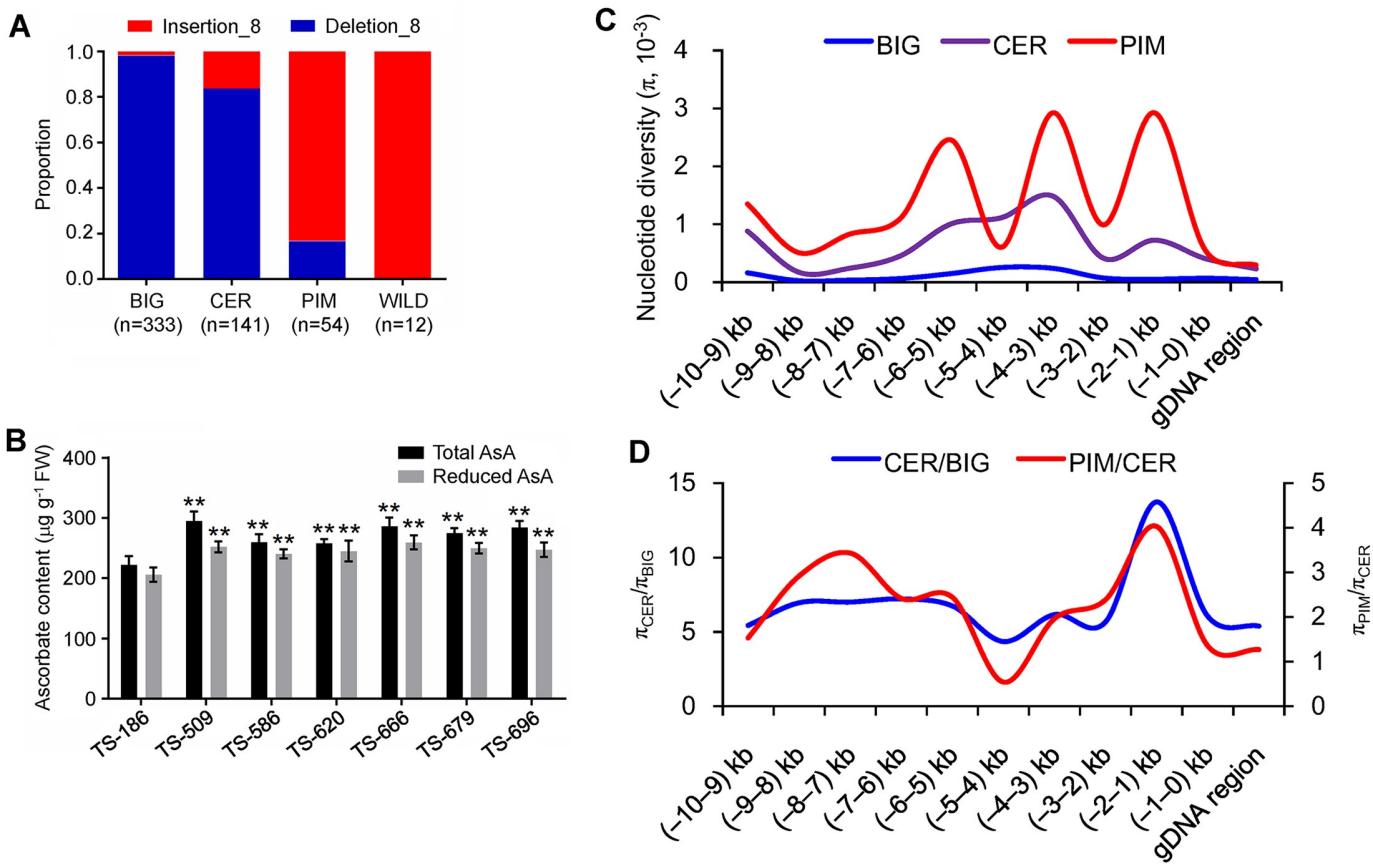
Nucleotide diversity analysis of *TFA9* loci during tomato domestication and improvement. **(A)** Frequency of derived and ancestral InDel_8 allele in tomato subpopulations. n = number of accessions. **(B)** Comparison of ascorbate content of Hap 1 accession TS-186 and six Hap 4 accessions in BIG subgroup. The data are presented as means ± SE (n = 3). The asterisks represented significant differences from TS-186 (Hap 1), as indicated by the *t*-test (** *P*< 0.01). **(C)** Distribution of nucleotide diversity (π) of the PIM (red line), CER (purple line), and BIG (blue line) within the 10-kb region of *TFA9*. Grey lines indicate the locations of indel_8 (TCTCTTTC/-). The X-axis denotes the position of *SlbHLH59* and the Y-axis indicates average *p* values. **(D)** The ratio of nucleotide diversity (π) is calculated from the *SlbHLH59* sequence of PIM with CER (red line) and CER with BIG (blue line). In total, 331 tomato accessions were used for analysis, including 53 PIM accessions, 112 CER accessions, and 166 BIG accessions.

Since InDel_8 represents a functional polymorphism of the *TFA9* locus for AsA biosynthesis via *SlbHLH59* expression in tomato fruit, we investigated InDel_8 variants in 540 accessions, including 333 BIG, 141 CER, 54 PIM and 12 accessions of wild tomato species (Fig 6A and S13 Table). Only 6 accessions in the BIG group carried insertion_8, as TS-265, and all other accessions carried deletion_8, as TS-186. Significant differences in both total and reduced AsA were detected between the eight insertion_8 BIG accessions (BIG^insertion_8^) and TS-186 (BIG^deletion_8^) (Fig 6B). All 12 accessions of wild species carried insertion_8 but the deletion_8 was detected in 9 of 54 tested PIM accessions, suggesting the deletion of InDel_8 occurred in early domestication during the time the PIM group differentiated from wild species. Thirteen of the 23 CER^insertion_8^ carrying accessions originated from South America, including those from Ecuador and Peru where wild tomato relatives originated. Thus, it is likely that the deletion_8 initially occurred in South America and subsequently spread into other neighbouring countries.

The high fruit AsA-associated insertion_8 was present at a highest frequency of 83.3% (45/54) in wild progenitor variety PIM, but the frequency sharply decreased in CER accessions (16.3%, 23/141) that were domesticated from the wild progenitor variety PIM and BIG landraces (1.8%, 6/333) (Fig 6A). The result revealed that the less fruit AsA content was selected during domestication. To examine the evolutionary history of the *TFA9* locus, DNA sequence variation in the genomic region spanning 10 kb upstream fragment and coding region of the *SlbHLH59* was investigated. On average, the nucleotide diversity (π) in the coding and promoter region of *SlbHLH59* was much lower in BIG (π = 0.11×10^−3^) than in CER (π = 0.65×10^−3^) and PIM (π = 1.32×10^−3^) (Fig 6C). The highest ratios of nucleotide diversity in PIM to CER (π_PIM_/π_CER_, 4.03) and CER to BIG (π_CER_/π_BIG_, 13.74) occurred at -2 kb to -1 kb region of *SlbHLH59*, where the indel_8 was located on (Fig 6D). This result was consistent with previous report that *SlbHLH59* was pointed out as a domestication and improvement sweep at the whole genome level [26]. Moreover, Tajima’s *D* of the region from -2 kb to -1 kb was negative in BIG (-1.53) subgroup but positive in the CER (0.46) and PIM (2.33) subgroup, respectively (S14 Table). Taken together, these results suggested that *TFM6* region has been subjected to human selection during the domestication of tomato.

## Discussion

Plant breeders have long focused on traits with potential to increase yields while decreasing inputs [33]. More recently, improving nutritional and flavour quality are of economic and social interest to help meet nutritional security needs of an increasing human population [46, 47]. Despite the success in generating tomato varieties with improved traits [48, 49], the efficiency of genetic improvement of this crop has remained relatively limited [8]. Here we implemented GWAS to obtain a detailed understanding of the genetic determinants underlying metabolic variation in tomato with the ultimate goal of enhancing genetic improvement of nutritional and flavour. We measured 92 metabolic traits and identified 388 loci involved in their variation. Among the candidate genes involved in these loci, most were annotated as transporters or regulators as opposed to structural enzymes (S7 Table). Finally, we characterized a candidate gene, *SlbHLH59*, involved in the variation of ascorbate. Then, we characterized a major QTL, *TFA9*, underlying the variation of ascorbate. Finally, we characterized a candidate gene, *SlbHLH59* corresponding to *TFA9*, and found it contribute to the variation of ascorbate which improved both fruit nutrition and oxidative stress tolerance in tomato.

mGWAS on tomato fruit metabolic traits has been conducted previously [28, 50]. Sauvage *et al*. (2014) preformed mGWAS using 39 primary metabolites and 5,995 SNPs among 163 tomato accessions, while 60 primary and secondary metabolites (including 33 volatiles) and 10,000 SNPs were used in the mGWAS by Bauchet *et al*. (2017). Together these led to identification of 44 and 79 loci significantly associated with 19 and 32 metabolic traits, respectively. We used over 4 million SNPs with 92 measured primary metabolites for genotype-phenotype association, leading to 388 highly suggestive loci for 75 metabolic traits (Table 1). For example, 17 associated signals of citric acid were identified (S5 Fig and S5 Table), as compared to up to 4 loci previously associated with citric acid [28, 50]. Four loci significantly associated with SSC including *Lin5* were also detected (S6 Fig), while only *Lin5* was previously reported [50]. We narrowed mapping resolution to a single gene in some cases. For example, a lead SNP on chr01 (ch01_79524657) located in a glutamine synthetase gene (*Solyc01g080280*) significantly associated with isoleucine content (S5 Table).

Unlike the GWAS results of secondary metabolites [51, 52], regulators and transporters rather than structural enzymatic genes involved in organic acid and sugar metabolism were identified (S7 Table). We hypothesized that the transportation and regulation of sugar and organic acids synthesis harbors more exploitable genetic variation than its synthesis in tomato fruits, consistent with previous studies [3, 27, 31, 32]. Two candidate genes, *SlbHLH59* as a regulator of ascorbate and *SlSTP11* as a transporter of SSC, were functionally identified to support this hypothesis. Previous studies have shown that the accumulation of AsA does not correlate with the expression of genes involved in its biosynthesis [53]. *SlbHLH59*, located at the end of chromosome 9, was associated with fruit ascorbate content consistent with previous reports by linkage mapping [36]. Notably, the regulatory role of bHLH transcription factor, *SlbHLH59*, involved in the biosynthesis of AsA was firstly reported in tomato. Given that the expression of *SlbHLH59* was associated with ascorbate content in different accessions, we concluded that the InDel_8 in the promoter of *SlbHLH59* play an important role in determining natural AsA variation and was selected during domestication (S11 Fig).

Taking advantage of this valuable resource for tomato genetic and metabolic variation, we uncovered the genetic basis underlying the variation in primary metabolism among subgroups of our diverse collection. This information could be directly used to help design breeding strategies for the improvement of high-value metabolites. Although a more complex genetic architecture has been revealed for primary metabolism compared with secondary metabolism [21], the considerable number of metabolites with major loci (*R*^*2*^>15%), suggest that breeding efforts for some metabolites can be simplified by pyramiding favourable alleles of major genes. Moreover, in addition to *SlbHLH59* that was verified by transgenic lines, hundreds of additional loci identified in this study remain to be fully explored to help dissect the molecular basis of metabolic variation in tomato. Further evaluation and validation of polymorphisms as was done for *SlbHLH59* should help uncover the genetics of natural variation in primary metabolism and expand the crop breeding toolbox for important fruit traits.

## Methods

### Plant materials and growth conditions

A total of 302 tomato accessions, including 171 *Solanum lycopersicum* (BIG), 104 *S*. *lycopersicum*var.*cerasiforme* (CER) and 27 *S*. *pimpinellifolium* (PIM) accessions that were selected from the previously described 360 accessionsand used for GWAS in this study. The GWAS was conducted at two sites: E1 (Spring 2013, open field at Huazhong Agricultural University, Wuhan, China) and E2 (Spring 2016, greenhouse at the Agri-Academy of Sciences of Wuhan, China). A F_2_ population of 1,587 individuals was derived from a cross between TS-21 (high SSC) and HG22 (low SSC) conducted in an open field at Huazhong Agricultural University in spring 2014. For GWAS and BSA, at least three fruits from at least three plants per line were harvested at the ripe stage. For fruit development analysis, flowers were tagged at the full-bloom stage to synchronize developmental stages. The fruits were harvested at immature green (IMG, 21 DAF), mature green (MG, 37 DAF), breaker (BR, 40 DAF), yellow ripe (YR, 42 DAF), and red ripe (RR, 49 DAF) developmental stages. Three biological replicates of each developmental stage were analyzed. After tissue selection, the outer pericarp was bulked (five fruits) and stored at –80°C for metabolic and transcript profiling. The remaining fresh red fruits were used directly to measure SSC, total sugars, total acids, and sugar/acid ratio.

### Sample preparation and metabolite profiling

Red fruit metabolite profiling of the 302 tomato accessions was performed by GC-MS using the method as described previously [27]. Tomato fruit juice samples were used to measure the SSC, total sugars, total acids, and sugar/acid ratio. SSC was determined within the GWAS population and F_2_ segregation population using a hand saccharimeter (B429335, ATAGO). The total sugar and total acid contents were measured using a Brix-Acidity Meter (PAL-BX|ACID3, ATAGO), followed by calculation of sugar/acid ratios. Total sugars and SSC in the fruit juice were measured by directly dropping the sample onto the meter and recording the value. The fruit juice was then diluted 50-fold with ddH_2_O water and used to determine total acids. The AsA levels were measured as previously described [44].

### Data processing and statistical analyses

The relative content of each metabolite was obtained by comparing the peak area of each metabolite with the peak area of internal standard (ribitol). For each phenotype, normal distribution of the data was tested using a Shapiro-Wilk test. The normality test revealed that 34 of the 92 phenotypes (37%) were not normally distributed and were Box-Cox transformed. The coefficient of variation values was calculated independently for each metabolite (using the mean of the biological replicates of the untransformed m-trait data) as follows: σ/μ, where σ and μ are the s.d. and mean of each metabolite in the population, respectively. Broad-sense heritability (*H*^*2*^) was calculated using the following equation by treating accessions as a random effect and the biological replication as a replication effect using one-way ANOVA: *H*^*2*^ = Var_(G)_ /(Var_(G)_ + Var_(E)_), where Var_(G)_ and Var_(E)_ are the variance derived from genetic and environmental effects, respectively. Finally, differences in the metabolic traits among the six subgroups (PIM, SA_CER, NSA_CER, UO_CER, NP_BIG, and P_BIG) were analysed by ANOVA tested. Significance was declared at *P* <0.05. The LD heatmap surrounding candidate gene in the GWAS was constructed using Haploview 4.2 with default parameters [54], indicating *r*^*2*^ values between pairs of SNPs multiplied by 100.

### Association mapping

To facilitate SNP identification and genotype imputation, two sequencing data sets were used in this study. The first was downloaded from a diverse global collection of 360 tomato accessions (NCBI Sequence Read Archive [SRA] under accession number SRP045767) [26]. Additional sequence data from 398 varieties were generated by Tieman *et al*. [20] was downloaded from the National Center for Biotechnology Information BioProject site under accession number PRJNA353161. SOAP2 was used to map all sequencing reads from each accession to the tomato reference genome (Version SL2.50) with the following parameters: -m 100, -x 888, -s 35, -l 32, -v 3. Mapped reads were filtered to remove PCR duplicates. Both paired-end and single-end mapped reads were used for SNP calling throughout the entire collection of tomato accessions using SOAPsnp with the following parameters: -L 100 -u -F 1(23). After imputation, SNPs with missing rates of less than 20% were selected, resulting in a total of 4,180,023 SNPs (MAF > 0.05, the number of varieties with the minor allele ≥ 6) for further analysis. Detailed information on called SNPs can be viewed and downloaded from Sol Genomic Network (https://solgenomics.net/).

Association analyses were performed using the compressed MLM [55] with TASSEL 4.0 [56]. Suggestive (1/n, ≤2.4×10^−7^) and significant (0.05/n, ≤1.2×10^−8^) *P*-value thresholds were defined to control the genome-wide type 1 error rate (n = total number of markers used) [51, 57]. We used Haploview software to perform local LD analysis [54] and calculate LD accordingly with modification [20]. Briefly, the average linkage decay for each 0.5 Mb region of the whole genome was evaluated with the following parameters: -maxdistance 2000 -minMAF 0.05 -hwcutoff 0. Pairwise LD between the suggestive/significant SNPs for each metabolic trait was calculated. The physical locations of the SNPs were identified based on tomato genomic sequence version SL2.50 (http://solgenomics.net/).

### Linkage mapping

An F_2_ population of 1,587 individuals derived from a cross between TS-21 (a high-SSC accession) and HG22 (a low-SSC accession) was planted in the Spring of 2014 in an open field at Huazhong Agricultural University, China. For each individual, the average SSC of three representative fruits was recorded, and genomic DNA was isolated from fresh leaves using the CTAB method. For bulked segregant analysis, bulk DNA samples for high- and low-SSC accessions were constructed by mixing equal amounts of DNA from 50 individuals showing extremely high and low SSC, respectively. Subsequently, 34.97× genome sequences for TS-21, 35.56× genome sequences for HG22, and roughly 60× genome sequences for each bulk sample (high-SSC fruit and low-SSC fruit) were generated by BIOMARKER Company (Beijing, China). The SLAF (Specific-Locus Amplified Fragment) label was located on the reference genome using SOAP, and labels that were sequenced <5× in the parent were filtered out. Short reads were aligned against the reference genome (release SL2.50) using the Burrows-Wheeler Aligner (BWA). The ΔSNP index was obtained by subtracting the SNP index of the low-SSC bulk sample from that of the high-SSC bulk sample. The average SNP index for the high-SSC and low-SSC bulk samples was calculated using a 1,000-kb sliding window with a step size of 10 kb. The statistical confidence intervals of the ΔSNP index were calculated under the null hypothesis of no QTLs, and 0.32 was then set as the threshold.

### Epistasis analysis

For each metabolic trait in each environment, the pairwise additive-by-additive epistatic interactions were investigated for all identified loci. Epistatic interactions were determined by two-way analysis of variance (ANOVA) (using *P*< 0.05 as a significance threshold) using all significant loci in pairwise combinations. The proportion of variance explained by epistasis was tested by comparing the residual of the full model containing all single-locus effects and two-locus interaction effects with that of the reduced model containing all single-locus effects but excluding two-locus interaction effects [58].

### DNA sequencing

To detect the variation in *SlbHLH59* gene region (chromosome 9: 64,094,000–64,101,000, release SL2.50), DNA sequences of *SlbHLH59* in 30 tomato accessions (15 high-AsA and 15 low-AsA accessions) were amplified by PCR using primers listed in (S15 Table). The PCR products were sequenced and compared against the reference genome for polymorphism analysis. In addition, DNA sequences of *SlbHLH59* in 367 tomato accessions (250 BIG accessions, 94 CER accessions and 23 PIM accessions) and the data for genotype analysis of InDel_8 in 540 tomato accessions were downloaded from the public database (National Center for Biotechnology Information BioProject site under the accession PRJNA353161).

### Gene cloning, vector construction, and transformation

For overexpression construct, completed *SlbHLH59* open reading frame (ORF) were amplified from the cDNA of tomato (TS-186 for *SlbHLH59*^SNP9A^ and TS-265 for *SlbHLH59*^SNP9G^), and then incorporated into the pHELLSGATE8 vector using homologous recombination (ClonExpress II One Step Cloning Kit, Vazyme). For RNAi construct, a 200-bp fragment of *SlbHLH59* was amplified by *SlbHLH59*-RI primers (S15 Table), and then cloned into the pHGRV vector using BP Clonase according to the manufacturer’s instructions (Invitrogen, USA). All the recombinant constructs were transformed into *Agrobacterium* strain C58 by electroporation and subsequently transformed into the tomato genome (TS-186 for overexpression and Ts-265 for RNAi) using cotyledon explants as described previously [59]. Transgenic plants were confirmed by PCR using CaMV35S promoter forward primer and *SlbHLH59* specific reverse primer (S15 Table).

### RNA isolation and gene expression analysis

Total RNA was extracted from different accessions and transgenic lines using TRIZOL reagent (Invitrogen, USA). Gene expression was investigated by qRT-PCR. The primer pair sequences (designed using Primer Premier 3.0 [http://frodo.wi.mit.edu/primer3]) are listed in S15 Table. The cDNA synthesis and qRT-PCR steps were performed as previously described [60]. The *Actin* gene (Solyc11g008430) was used as an internal standard and qRT-PCR was performed with three repeats per gene (including *ACTIN*).

### Subcellular localization

The coding sequences of SlbHLH59^SNP9A^and SlbHLH59^SNP9G^ without the stop codon was amplified from the cDNA of tomato (TS-186 for *SlbHLH59*^SNP9A^ and TS-265 for *SlbHLH59*^SNP9G^) by PCR and then cloned into the expression vector p101YFP under the control of the CaMV35S promoter by homologous recombination. CaMV35S: SlbHLH59-YFP vector as well as cell nucleus marker CaMV35S: ERF-YFP was transformed into *Agrobacterium tumefaciens* strain GV3101 and co-infiltrated into leaves of *N*. *benthamiana* with the suspension as previously described [61]. After 48 h incubation at 25 °C, the tobacco leaves were used for YFP and RFP fluorescence signal observation using Leica Confocal software. CaMV35S:YFP acted as positive control.

### Yeast one-hybrid assay

The yeast one-hybrid assay was performed as described in the Matchmaker One-Hybrid Library Construction and Screening Kit (Clontech). Briefly, the full-length of *SlbHLH59* ORF sequence (amplified from TS-186 cDNA) and promoter sequences of *SlPMM*, *SlPMI*, *SlGME1*, *SlGMP1*, *SlGMP2*, *SlGMP3* and *SlGMP4* (amplified from TS-186 genomic DNA) were cloned into the pGADT7 and pAbAi vector (Clontech), respectively. The pAbAi bait vectors were introduced into the GOLD1 yeast and cultured on SD/–Ura. The pGADT7 prey vector was introduced into yeast strains containing pAbAi bait vectors and cultured on SD/–Leu. After 4 d incubation, the positive yeast strains were picked and diluted in double-distilled water to an OD600 of 0.1, and 2 μL of suspension was spotted on SD/–Leu, with or without ABA (0–20 ng/mL) (Sigma-Aldrich), followed by 3 to 7 d incubation at 30°C.

### Transient expression in *N*. *benthamiana* leaves and *Arabidopsis* protoplasts

The full-length *SlbHLH59* ORF was cloned into the effector vector pGreen II 62-SK under the control of CaMV 35S promoter. *SlPMM*, *SlGMP2* and *SlGMP3* promoter fragments were PCR amplified using specific primers and cloned into the reporter vector pGreen II 0800-LUC. Individual combinations of effector and reporter vectors were co-transformed into *Agrobacterium* GV3101 cells alongside the pSoup vector, and the transformed GV3101 cells were used to infiltrate young *N*. *benthamiana* leaves, in which transient expression was analyzed following a 2-d incubation. Firefly and Renilla luciferase signals were assayed with the dual luciferase assay reagents (Promega) using an Infinite M200 (Tecan).

The promoter activity analysis was carried out as described previously [62]. GUS activity and LUC activity were determined by Fluorescence FLx800 microplate fluorescence reader (BIO-TEK Instruments). Ratios of GUS to LUC activities were used to define relative promoter activity. Three biological replicates were performed for each construct. The *cis*-element analysis was conducted in PLACE (http://www.dna.affrc.go.jp/PLACE).

### Light response and oxidative stress treatment

One-month-old seedlings of*SlbHLH59*^*SNP9A*^ overexpression plants as well as TS-186 and TS-265 plants were grown in plastic pots in the greenhouse. For the light response characterization, from 8 am, the plants were exposed to continuous light at 25°C for 12 h followed by 12 h continuous dark under 25°C and recovered by12 h continuous light at 25°C. In a 36-h photoperiod, samples were taken every four hours (8:00, 12:00, 16:00 and 20:00 under light, 24:00 and 4:00 under light) to determine the expression of *SlbHLH59* in tomato leaves.

To evaluate the performance of *SlbHLH59*^*SNP9A*^ overexpression plants and wild type (TS-186) plants under oxidative stress, the plants were sprayed with 75 μM MV (MV dissolved in water with 0.1% Tween-20) or water with 0.1% Tween-20 (control) once a day for 2 days. Phenotype was investigated and recorded one week after the end of the treatment. For the 3–3’-diaminobenzidine (DAB) staining, the leaves were cleaned and placed in 1 mg/mL DAB, pH 3.8, under light at 25°C for 8 h. The experiment was terminated by immersing the leaves in boiling 96% ethanol for 10 min. After cooling, the leaves were placed in fresh 96% ethanol for 4 h at room temperature and photographed. The deep brown polymerization product was produced via the reaction of DAB with H_2_O_2_. Also, leaves were collected and ground into fine powder in liquid nitrogen after the treatment. To assay chlorophyll levels, 1 ml of 80% (v/v) acetone was added to approximately 0.1 g of frozen powder in a 2-ml Eppendorf tube under low light intensity by the procedure described by Wellburn [63]. The MDA levels were measured as previously described [64].

### Molecular diversity analysis

For the molecular diversity analysis, the π ratios and Tajima’s *D* [65], were used to identify the selective sweeps in *SlbHLH59* associated with tomato domestication and improvement events. Briefly, π (π_PIM_, π_CER_ and π_BIG_) and Tajima’s *D* (Tajima’s *D*_PIM_,Tajima’s *D*_CER_ and Tajima’s *D*_BIG_) were calculated using DnaSP5.0 version 5.0 software [66], with a sliding window length of 100 bp and step size of 25 bp.

## Supporting information

S1 Fig The coefficients of variation (*CV*) and broad-sense heritability (*H*^*2*^) for each metabolite. **(A)** Distribution of the genetic coefficients of variation (*CV*) of metabolic traits (*n* = 92); **(B)** Distribution of broad-sense heritability (*H*^*2*^) of metabolic traits (*n* = 49) detected in the association panel across the two environments.
(TIF)

S2 Fig Phenotypic distribution of 13 metabolic traits in different tomato subgroups. Phenotypic distribution of metabolic traits that were detected in both years (2013 in red, 2016 in blue) for the subgroups *S*. *pimpinellifolium*, PIM; *S*. *lycopersicum*var.*cerasiforme*, SA_CER, NSA_CER and UO_CER; *Solanumlycopersicum*, P_BIG and NP_BIG (see Methods). **(A)** Sugars, **(B)** organic acids, **(C)** amino acids, **(D)** three flavour-related metabolic traits (SSC, total sugars and total acids). For the box plot, the horizontal lines in boxes indicate the median values, the box height indicates the 25^th^ to 75^th^ percentile of the total data, the whiskers indicate the interquartile range, and the outer dots indicate outliers.
(TIF)

S3 Fig Suggestive loci (*P* < 2.4 × 10^−7^) for the GWAS results from the two environments. Heatmap displaying the GWAS results for 73 metabolites with significant loci. The *x*-axis indicates the genomic locations by chromosomal order. The significant loci (-log_10_*P*) are plotted against the genome location in 200-kb intervals. Each row represents one metabolite. Detailed information for all detected loci is shown in S5 Table. Metabolites from different groups are marked with different colours, as shown on the right. m1, Alanine; m2, L-Lysine; m3, Valine; m4, Acetamide; m5, Asparagine; m6, Glycine; m7, Isoleucine; m8, L-Aspartic acid; m9, L-Cysteine; m10, L-Glutamic acid; m11, l-Glutamine; m12, L-Hydroxylysine; m13, L-Serine; m14, L-Threonine; m15, Phenylalanine; m16, 4-hydroxyproline; m17,Serine; m18, β-Alanine; m19, 3-Aminoisobutyric acid; m20, Acetic acid; m21, Total acids; m22, Aminobutyric acid; m23, Ascorbate; m24, Benzoic acid; m25, Citric acid; m26, D-Glucopyranosiduronic acid; m27, Docosatetraenoic acid; m28, Fumaric acid; m29, Galacturonic acid; m30, Glutaric acid; m31, Glycyl-l-glutamic acid; m32, Lactic acid; m33, L-Threonic acid; m34, Malic acid; m35, Octadecadienoic acid; m36, Palmitic acid; m37, Phosphate acid; m38, Pyridine-3-carboxylic acid; m39, Pyrimidinetrione; m40, Quininic acid; m41, Ribonic acid; m42, Stearic acid; m43, Succinic acid; m44, Timonacic; m45, 1,3-Propanediol; m46, 2-Amino-2-methyl-1,3-propanediol; m47, 2-Chloroethanol; m48, Amine; m49, Ethanolamine; m50, Galactose oxime; m51, Glycerol monostearate; m52, Hexopyranose; m53, L-5-Oxoproline; m54, Monolinoleoylglycerol; m55, Pentasiloxane; m56, Phenyl hydroxide; m57, Piperidine; m58, Sugar/Acid; m59, SSC; m60, Tricarbomethoxyethylene; m61, Arabinofuranose; m62, D-Glucopyranoside; m63, Fructose; m64, Galactose; m65, Gluconic acid sodium salt; m66, Glucose; m67, Mannobiose; m68, Mannose; m69, Myo-inositol; m70, Sucrose; m71, Sugar; m72, Xylose.
(TIF)

S4 Fig Epistatic interactions between significant loci for each metabolic trait. Proportion of phenotypic variation explained by all single QTLs and epistatic interactions was shown.
(TIF)

S5 Fig The GWAS results (A) and Quantile-Quantile (Q-Q) plot (B) of *P*-values for all traits mentioned in S7 Table. (TIF)

S6 Fig Functional identification of Solyc06g054270 (sugar transporter gene, STP11). **(A)** Manhattan plot displaying the GWAS results for fruit SSC (CMLM, N = 302). Negative log10-transformed P values from the compressed mixed linear model are plotted on the y-axis. Horizontal dashed line indicates a genome-wide significance threshold of 2.4×10−7. **(B)** Quantile-quantile plot for SSC in the GWAS population. **(C)** Detailed plot is shown for region 37–37.15 Mb on chromosome 6 (x-axis). Lead SNP is indicated in purple. A representation of pairwise r2 values (a measure of LD) among all SNPs in 37–37.15 Mb, where the colour of each box corresponds to the r2 value according to the legend. **(D)** SSC in TS-21, HG22, and their F1 progeny. **(E)** The frequency distribution of fruit SSC in F2 progeny from a cross between TS-21 and HG22. Arrows indicate fruit SSC in the parental accessions. **(F)** Box plot of SSC. L_SSC pool and H_SSC pool indicates the low SSC and high SSC pool, respectively. The two bulk populations with extreme SSC values from the F2 population each contain 50 individuals. **(G)** The ΔSNP index (determined by subtracting the SNP index of the L_SSC bulk population from that of the H_SSC bulk population). Horizontal dashed line indicates a significance threshold of 0.308. **(H)** Region with a ΔSNP index above the confidence line on chromosome 6. The position of Solyc06g054270 is marked with a red line. **(I)** Gene structure of STP11 and natural variation between alleles from TS-21 and HG22. */- marks InDels between TS-21 and HG22. **(J)** Relative levels of STP11 mRNA in TS-21 and HG22. Expression levels were measured by qRT-PCR, and the values for three biological replications were averaged (**P < 0.01; t test).
(TIF)

S7 Fig The relative expression of *Solyco9g065830* (*SlDHUFS*) in fruits of 30 tomato accessions. The expression of *Solyco9g065830* was investigated in fruits from fifteen low-AsA accessions and fifteen high-AsA accessions referred in Fig 2H and 2G. Data represent means ± s.d. (n = 3).
(TIF)

S8 Fig Phylogenetic tree analysis of *bHLHs* in different species. **(A)** The model of amino acid sequence of SlbHLH59. The conserved bHLH domain of SlbHLH59 is indicated at the region of 150aa-194aa. Arrow indicates the location of SNP9 which is the only nonsynonymous mutation in SlbHLH59. **(B)** Phylogenetic tree analysis of *bHLHs* in different species. Full-length sequences of *SlbHLH59* orthologs from various plants were collected following NCBI-BLAST (see **Supplemental Data Set 1**). The neighbor-joining tree was constructed using MEGA 5 software. Numbers indicate bootstrap support based on 1000 replicates. *Solanum lycopersicum* are indicated as red circles; *Arabidopsis thaliana* are indicated as green squares; *Oryza sative* L. are indicated as yellow diamond. The SlbHLH59 and its orthologs (UNE12) in *Arabidopsis thaliana* are indicated.
(TIF)

S9 Fig The role of SlbHLH59 in light response. The expression of *SlbHLH59* in leaves of high AsA accession TS-265 and low AsA accession TS-186 were detected. The light green indicated that the tomato plants were in the light, while the light purple indicated plants in the dark.
(TIF)

S10 Fig The role of SlbHLH59 in oxidative stress. The chlorophyll **(A)** and malondialdehyde (MDA) **(B)** content in leaves were assayed 7 days after treatment with methyl viologen (MV) or water (CK). Three independent experiments were performed. The data presented are means ± SE. Asterisks represent significant differences from the control (CK), (*P < 0.05; **P < 0.01, *t*-test).
(TIF)

S11 Fig A possible regulation mechanism of *TFA9* in AsA metabolism during domestication of tomato. *SlbHLH59* promotes the biosynthesis of AsA by positively regulating the expression of structural genes (solid arrows and red marked genes mean the directly regulation of *PMM*, *GMP2* and *GMP3*; dotted arrows and black marked genes mean the indirectly regulation of *PMI*, *GPM1*, *GMP4* and *GME1*. The InDel_8 (green box) in the promoter of *SlbHLH59*, occurred during the tomato domestication and improvement and causes the present/absent of 5’ UTR Py-rich stretch motif, thus affects *SlbHLH59* the expression.
(TIF)

S1 Table Summary of the information of detected metabolites. (XLSX)

S2 Table Data for metabolic traits detected in the GWAS population in 2013. (XLSX)

S3 Table Data for metabolic traits detected in the GWAS population in 2016. (XLSX)

S4 Table Tomato accession groups with repeatedly detected traits subjected to pairwise difference tests (*t*-test). (XLSX)

S5 Table List of 388 detected suggestive SNPs (including 126 significant SNPs) in at least one environment. (XLSX)

S6 Table Epistatic interactions between significant loci of metabolic traits found to have multiple associated loci. (XLSX)

S7 Table Summary of 37 key candidate genes assigned from mGWAS results. (XLSX)

S8 Table Sequence polymorphisms between TS-21 and HG22 in the candidate gene STP11 identified by re-sequencing. (XLS)

S9 Table Genes within 100 kb of the SNP most highly associated with fruit ascorbate content. (XLSX)

S10 Table The SNPs and their *P* value in the haploblock20. (XLSX)

S11 Table Details of the four haplotypes of *SlbHLH59* in 367 tomato accessions. (XLSX)

S12 Table The number of E-boxes in promoter region of genes in ascorbate metabolism pathways. (XLSX)

S13 Table The genotype of InDel_8 in 540 tomato accessions. (XLSX)

S14 Table Nucleotide diversity analysis of *TFA9* during tomato domestication and improvement. (XLSX)

S15 Table Primers used in this study. (XLSX)

S1 File Amino acid sequences of 31 SlbHLH59 orthologs in plants referred to in S7 Fig. (DOCX)
