# Supplementary figures and images for: Genome-wide association analysis identifies a natural variation in basic helix-loop-helix transcription factor regulating ascorbate biosynthesis via D-mannose/L-galactose pathway in tomato
Source: PLoS Genet. 2019 May 8;15(5):e1008149. doi: 10.1371/journal.pgen.1008149 (PMC6527244; doi:10.1371/journal.pgen.1008149)

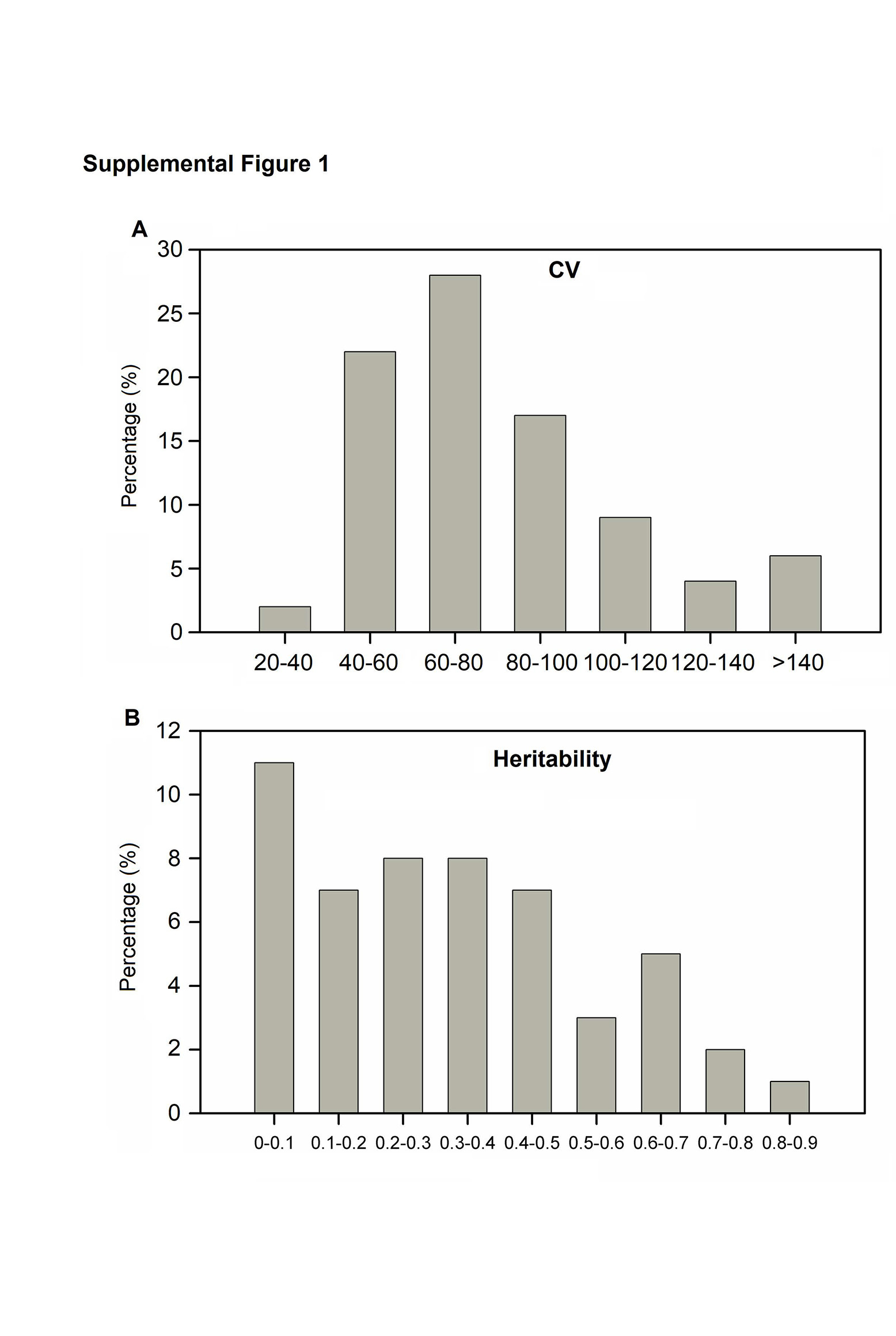

Supplement: S1 Fig — (A) Distribution of the genetic coefficients of variation (CV) of metabolic traits (n = 92); (B) Distribution of broad-sense heritability (H2) of metabolic traits (n = 49) detected in the association panel across the two environments. (TIF) [file pgen.1008149.s001.tif]

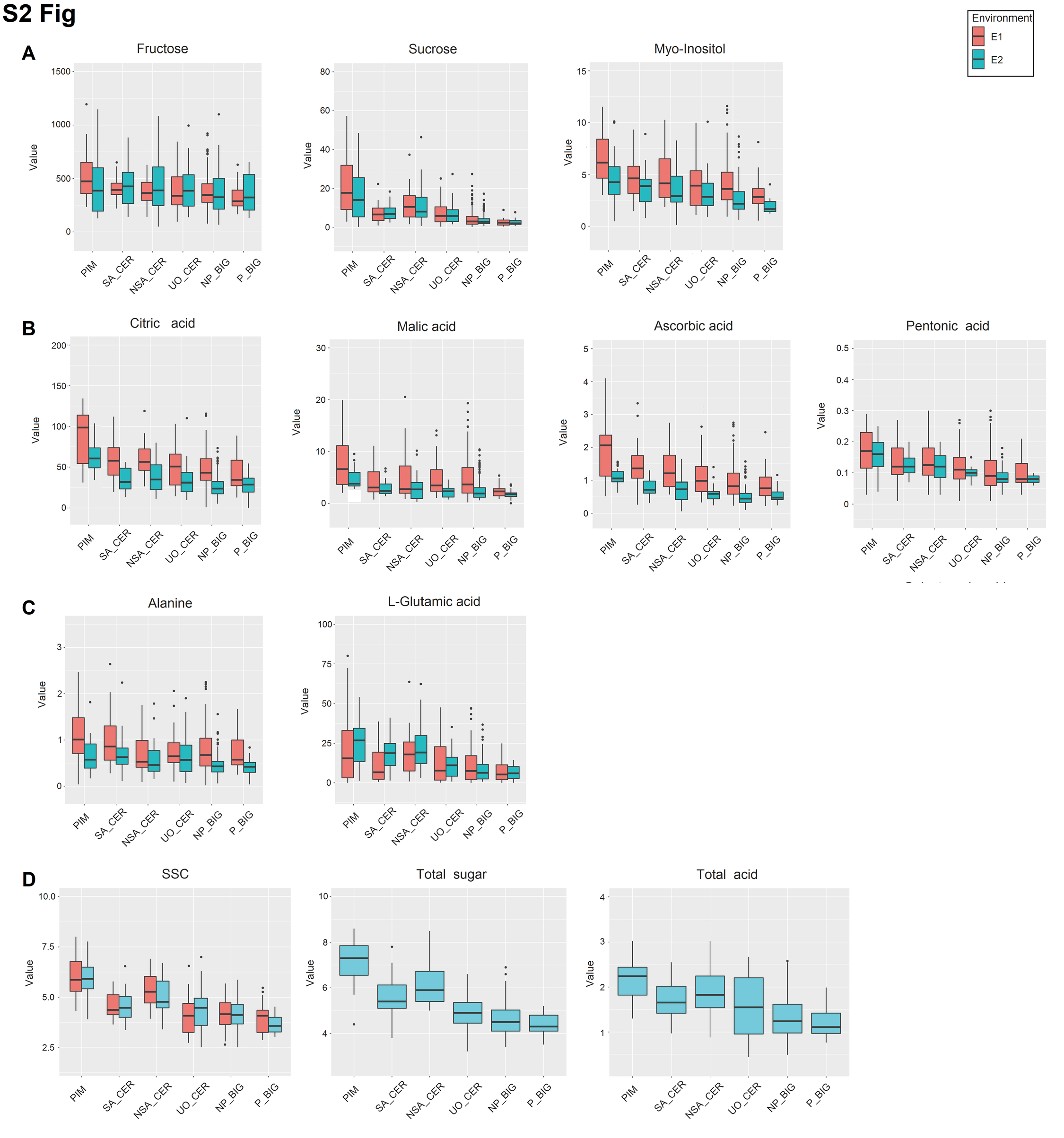

Supplement: S2 Fig — Phenotypic distribution of metabolic traits that were detected in both years (2013 in red, 2016 in blue) for the subgroups S. pimpinellifolium, PIM; S. lycopersicumvar.cerasiforme, SA_CER, NSA_CER and UO_CER; Solanumlycopersicum, P_BIG and NP_BIG (see Methods). (A) Sugars, (B) organic acids, (C) amino acids, (D) three flavour-related metabolic traits (SSC, total sugars and total acids). For the box plot, the horizontal lines in boxes indicate the median values, the box height indicates the 25th to 75th percentile of the total data, the whiskers indicate the interquartile range, and the outer dots indicate outliers. (TIF) [file pgen.1008149.s002.tif]

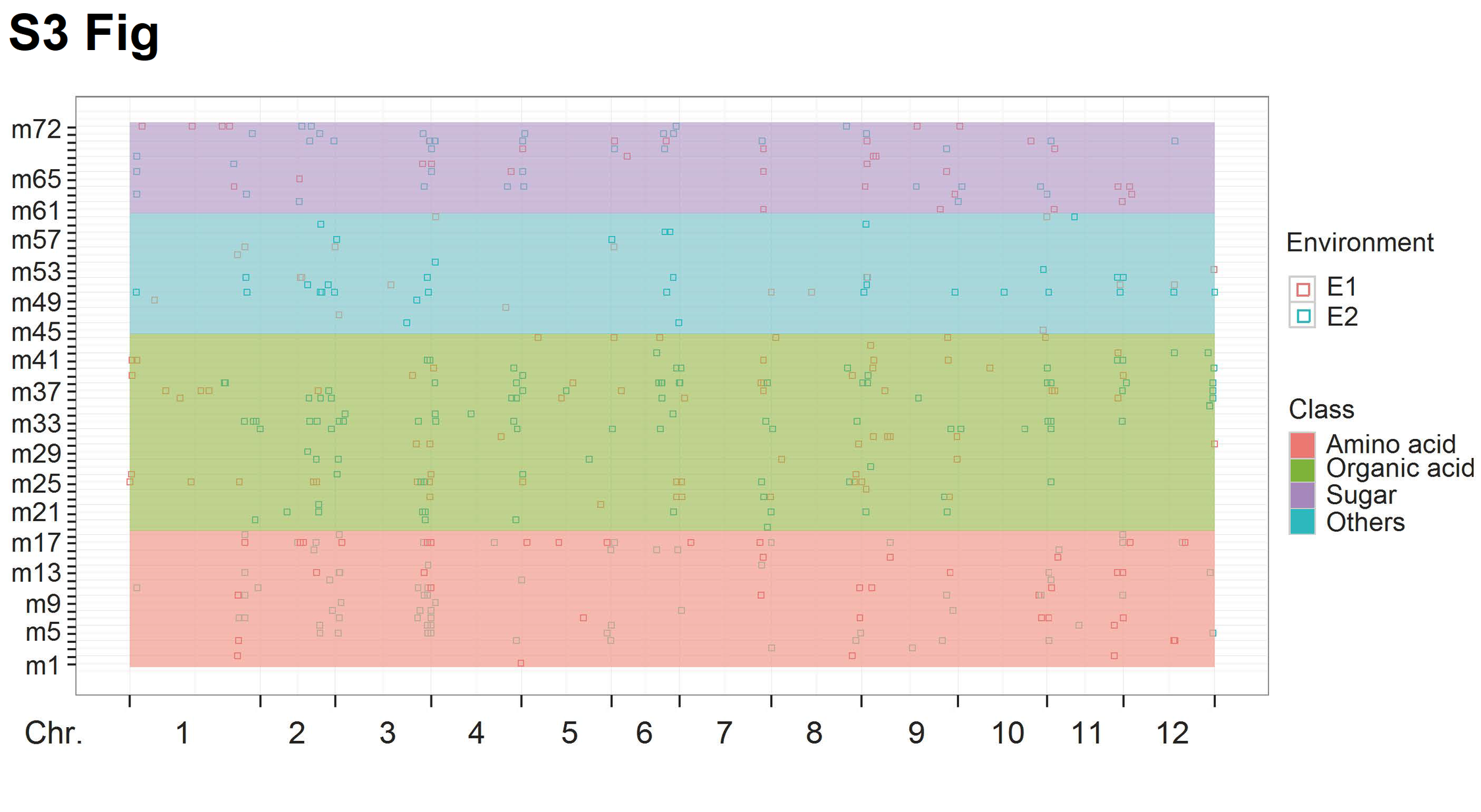

Supplement: S3 Fig — Heatmap displaying the GWAS results for 73 metabolites with significant loci. The x-axis indicates the genomic locations by chromosomal order. The significant loci (-log10P) are plotted against the genome location in 200-kb intervals. Each row represents one metabolite. Detailed information for all detected loci is shown in S5 Table. Metabolites from different groups are marked with different colours, as shown on the right. m1, Alanine; m2, L-Lysine; m3, Valine; m4, Acetamide; m5, Asparagine; m6, Glycine; m7, Isoleucine; m8, L-Aspartic acid; m9, L-Cysteine; m10, L-Glutamic acid; m11, l-Glutamine; m12, L-Hydroxylysine; m13, L-Serine; m14, L-Threonine; m15, Phenylalanine; m16, 4-hydroxyproline; m17,Serine; m18, β-Alanine; m19, 3-Aminoisobutyric acid; m20, Acetic acid; m21, Total acids; m22, Aminobutyric acid; m23, Ascorbate; m24, Benzoic acid; m25, Citric acid; m26, D-Glucopyranosiduronic acid; m27, Docosatetraenoic acid; m28, Fumaric acid; m29, Galacturonic acid; m30, Glutaric acid; m31, Glycyl-l-glutamic acid; m32, Lactic acid; m33, L-Threonic acid; m34, Malic acid; m35, Octadecadienoic acid; m36, Palmitic acid; m37, Phosphate acid; m38, Pyridine-3-carboxylic acid; m39, Pyrimidinetrione; m40, Quininic acid; m41, Ribonic acid; m42, Stearic acid; m43, Succinic acid; m44, Timonacic; m45, 1,3-Propanediol; m46, 2-Amino-2-methyl-1,3-propanediol; m47, 2-Chloroethanol; m48, Amine; m49, Ethanolamine; m50, Galactose oxime; m51, Glycerol monostearate; m52, Hexopyranose; m53, L-5-Oxoproline; m54, Monolinoleoylglycerol; m55, Pentasiloxane; m56, Phenyl hydroxide; m57, Piperidine; m58, Sugar/Acid; m59, SSC; m60, Tricarbomethoxyethylene; m61, Arabinofuranose; m62, D-Glucopyranoside; m63, Fructose; m64, Galactose; m65, Gluconic acid sodium salt; m66, Glucose; m67, Mannobiose; m68, Mannose; m69, Myo-inositol; m70, Sucrose; m71, Sugar; m72, Xylose. (TIF) [file pgen.1008149.s003.tif]

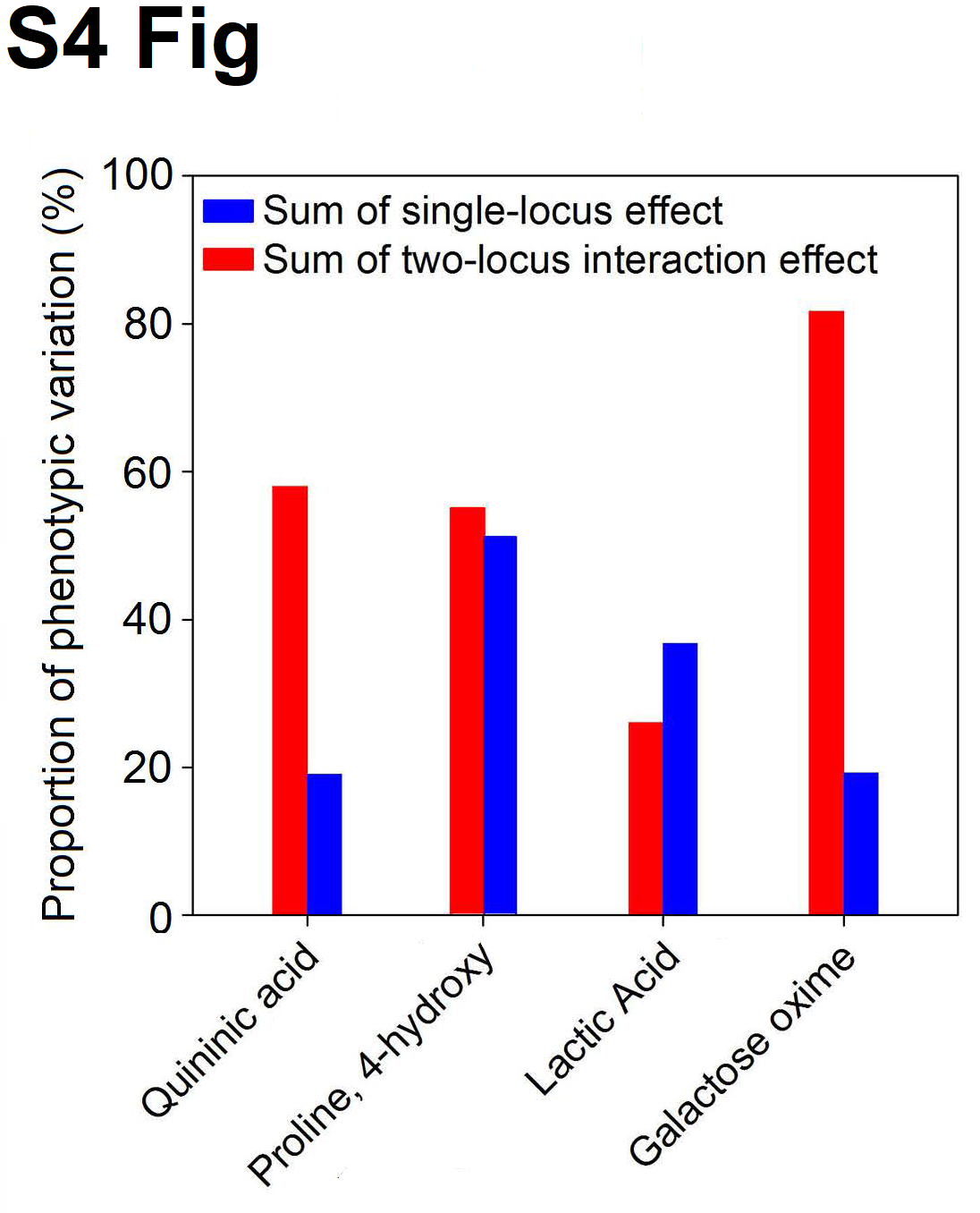

Supplement: S4 Fig — Proportion of phenotypic variation explained by all single QTLs and epistatic interactions was shown. (TIF) [file pgen.1008149.s004.tif]

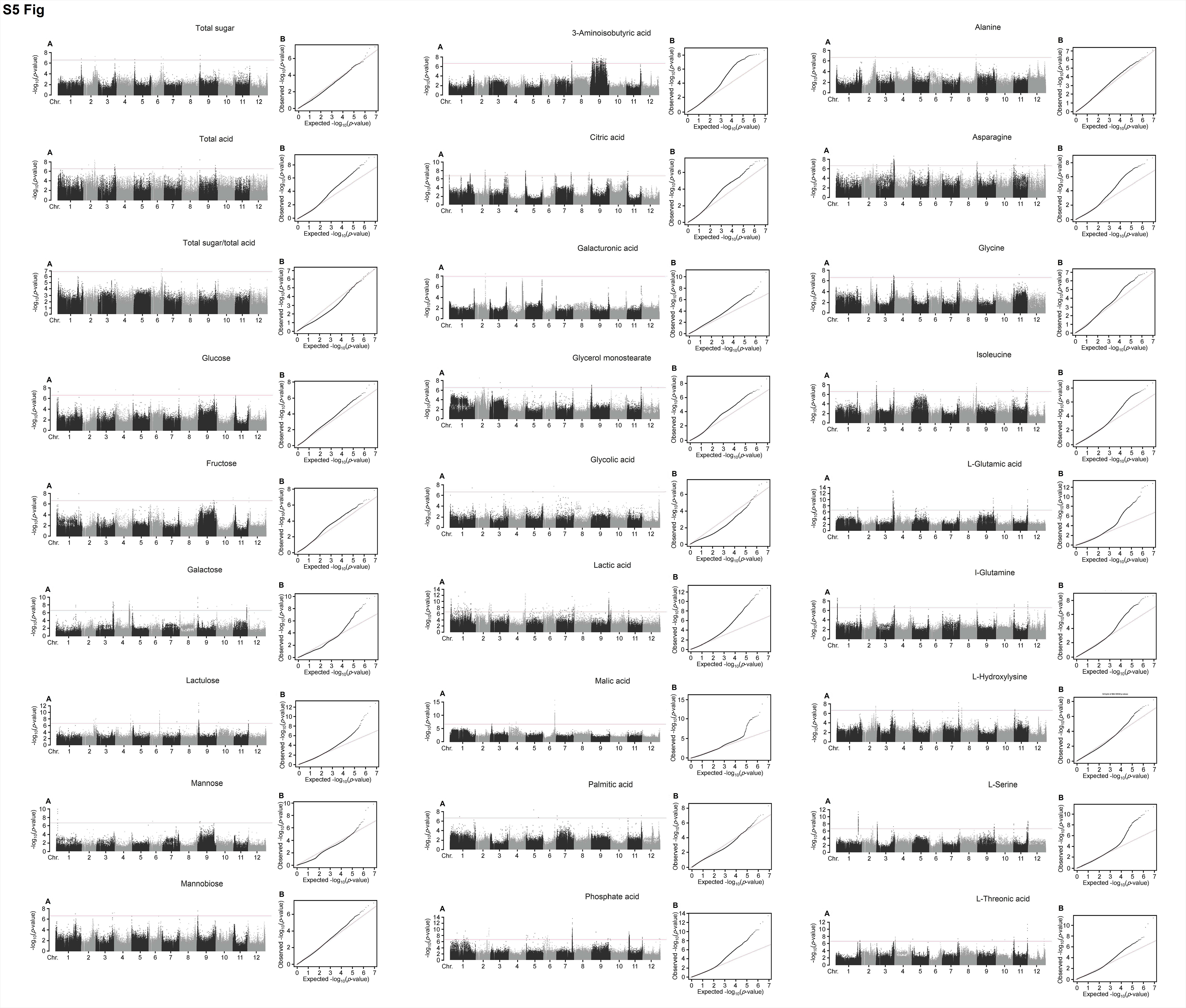

Supplement: S5 Fig — (TIF) [file pgen.1008149.s005.tif]

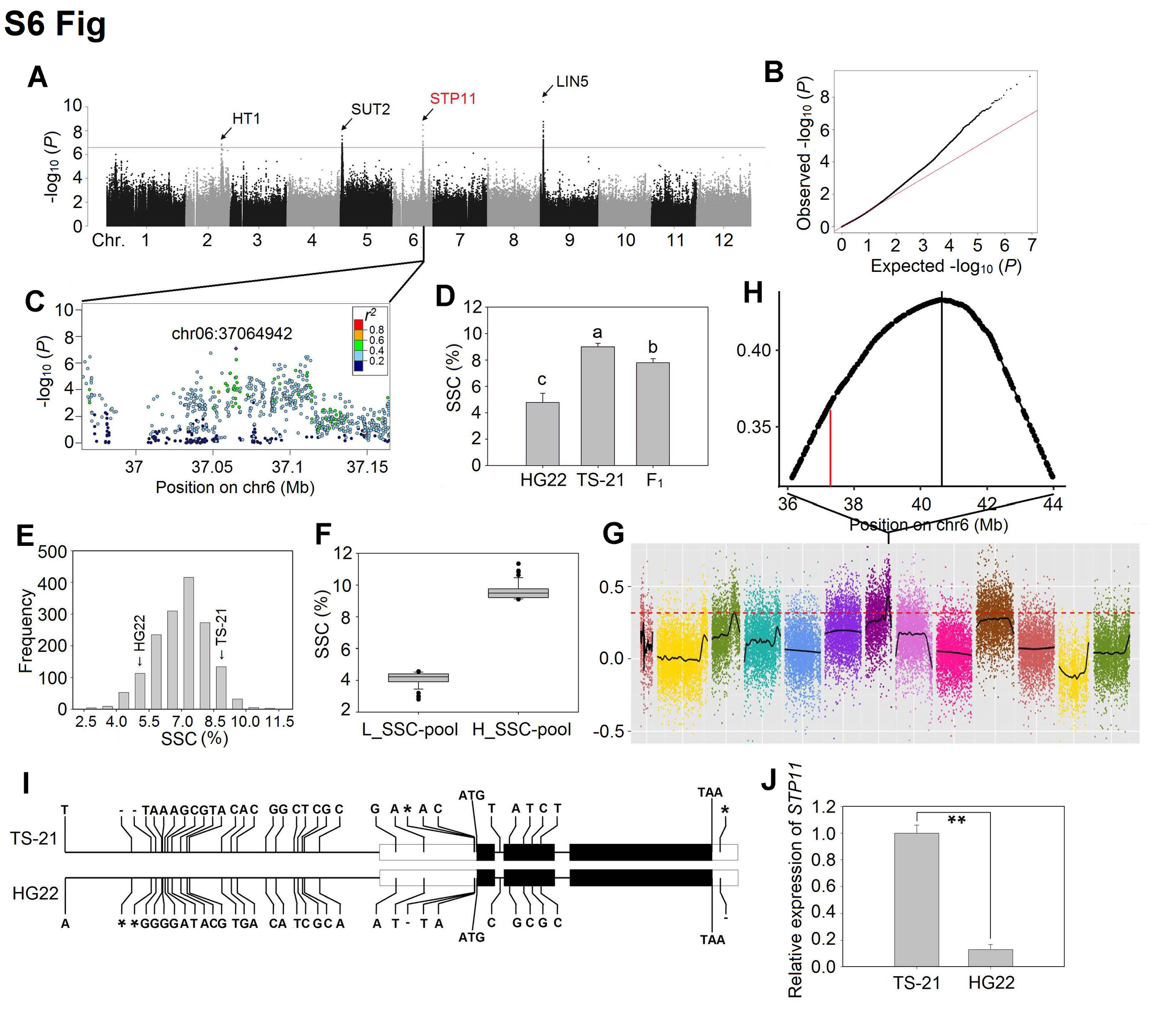

Supplement: S6 Fig — (A) Manhattan plot displaying the GWAS results for fruit SSC (CMLM, N = 302). Negative log10-transformed P values from the compressed mixed linear model are plotted on the y-axis. Horizontal dashed line indicates a genome-wide significance threshold of 2.4×10^−7^. (B) Quantile-quantile plot for SSC in the GWAS population. (C) Detailed plot is shown for region 37–37.15 Mb on chromosome 6 (x-axis). Lead SNP is indicated in purple. A representation of pairwise r2 values (a measure of LD) among all SNPs in 37–37.15 Mb, where the colour of each box corresponds to the r2 value according to the legend. (D) SSC in TS-21, HG22, and their F1 progeny. (E) The frequency distribution of fruit SSC in F2 progeny from a cross between TS-21 and HG22. Arrows indicate fruit SSC in the parental accessions. (F) Box plot of SSC. L_SSC pool and H_SSC pool indicates the low SSC and high SSC pool, respectively. The two bulk populations with extreme SSC values from the F2 population each contain 50 individuals. (G) The ΔSNP index (determined by subtracting the SNP index of the L_SSC bulk population from that of the H_SSC bulk population). Horizontal dashed line indicates a significance threshold of 0.308. (H) Region with a ΔSNP index above the confidence line on chromosome 6. The position of Solyc06g054270 is marked with a red line. (I) Gene structure of STP11 and natural variation between alleles from TS-21 and HG22. */- marks InDels between TS-21 and HG22. (J) Relative levels of STP11 mRNA in TS-21 and HG22. Expression levels were measured by qRT-PCR, and the values for three biological replications were averaged (**P < 0.01; t test). (TIF) [file pgen.1008149.s006.tif]

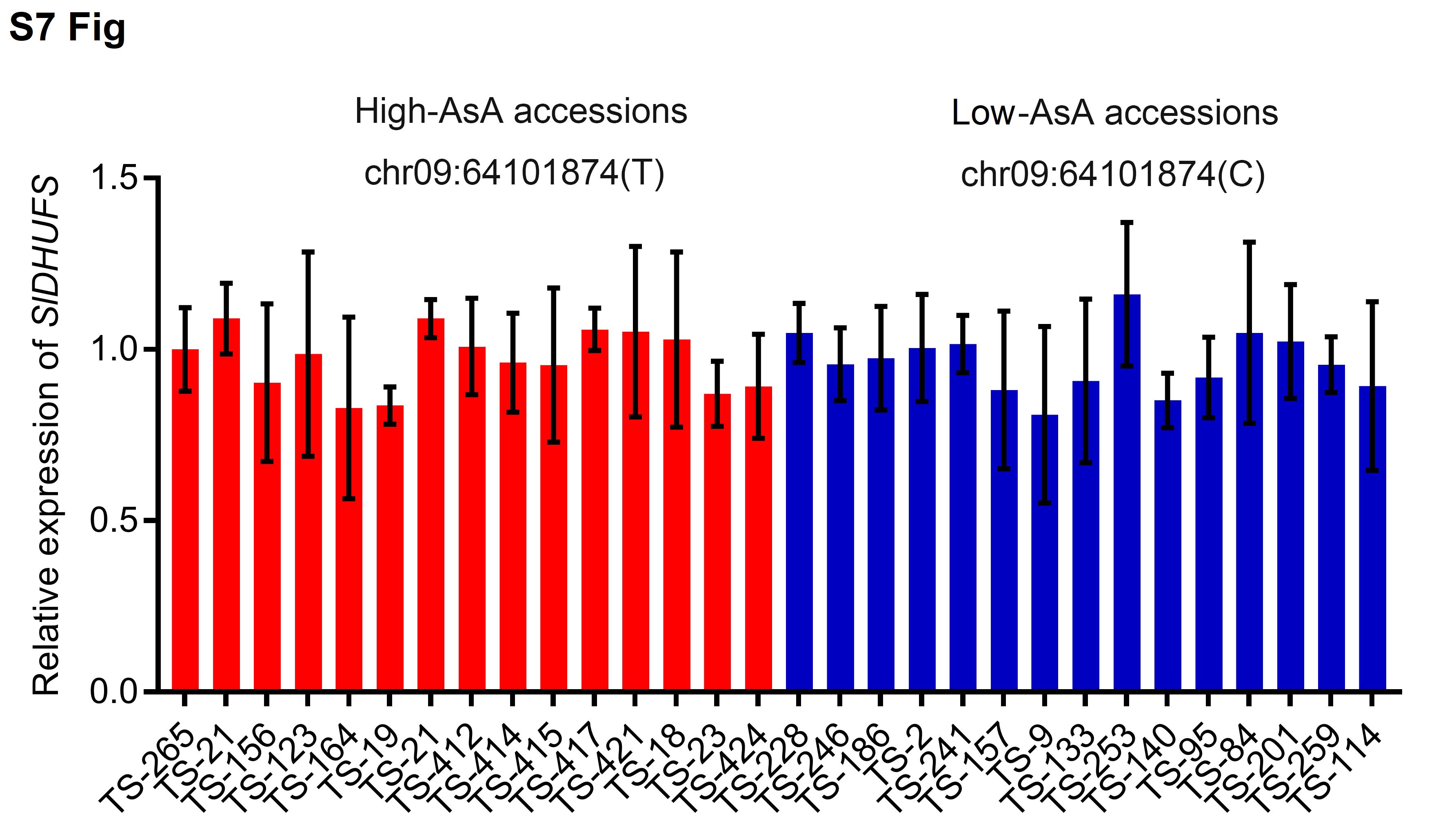

Supplement: S7 Fig — The expression of Solyco9g065830 was investigated in fruits from fifteen low-AsA accessions and fifteen high-AsA accessions referred in Fig 2H and 2G. Data represent means ± s.d. (n = 3). (TIF) [file pgen.1008149.s007.tif]

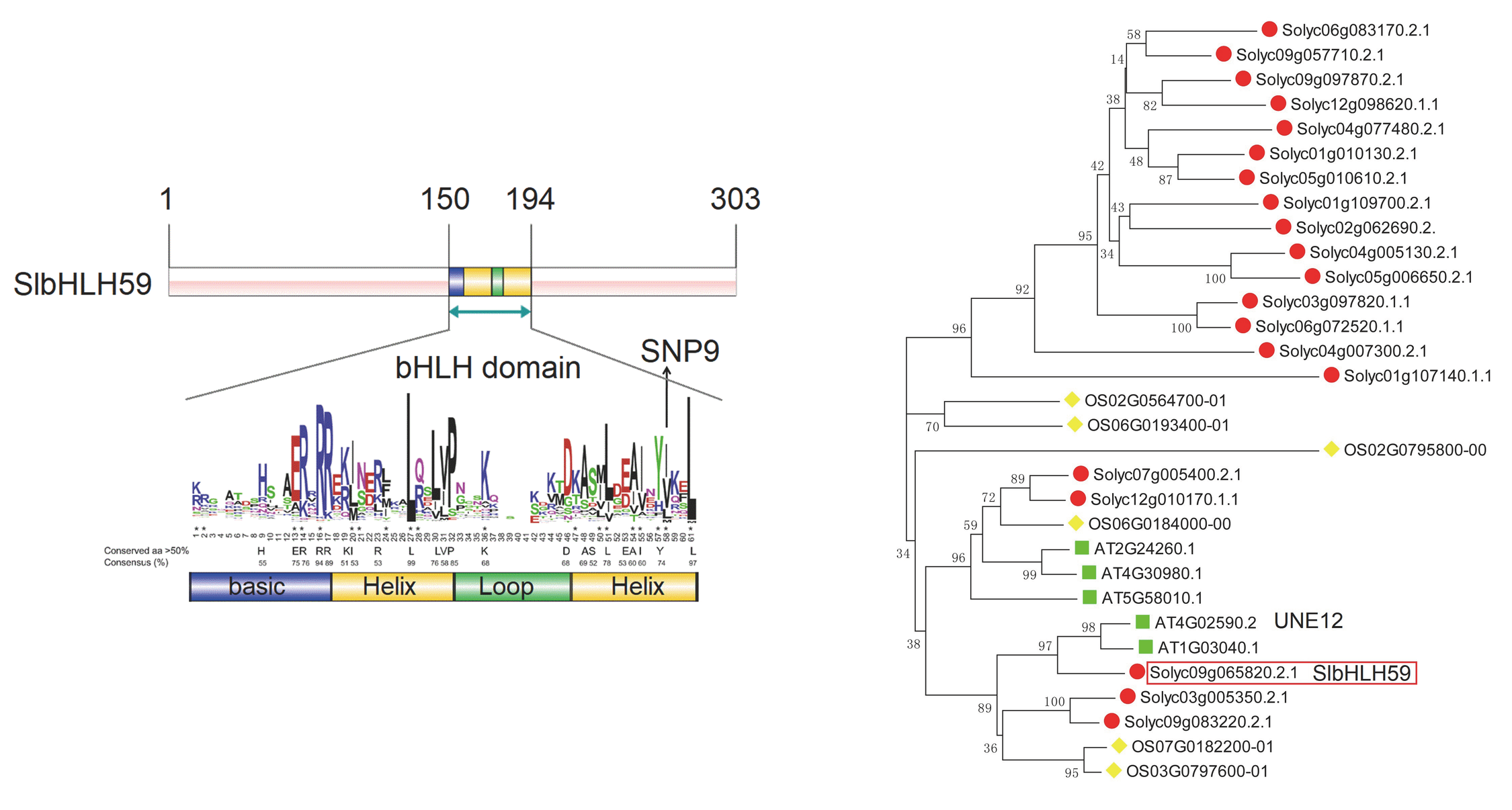

Supplement: S8 Fig — (A) The model of amino acid sequence of SlbHLH59. The conserved bHLH domain of SlbHLH59 is indicated at the region of 150aa-194aa. Arrow indicates the location of SNP9 which is the only nonsynonymous mutation in SlbHLH59. (B) Phylogenetic tree analysis of bHLHs in different species. Full-length sequences of SlbHLH59 orthologs from various plants were collected following NCBI-BLAST (see Supplemental Data Set 1). The neighbor-joining tree was constructed using MEGA 5 software. Numbers indicate bootstrap support based on 1000 replicates. Solanum lycopersicum are indicated as red circles; Arabidopsis thaliana are indicated as green squares; Oryza sative L. are indicated as yellow diamond. The SlbHLH59 and its orthologs (UNE12) in Arabidopsis thaliana are indicated. (TIF) [file pgen.1008149.s008.tif]

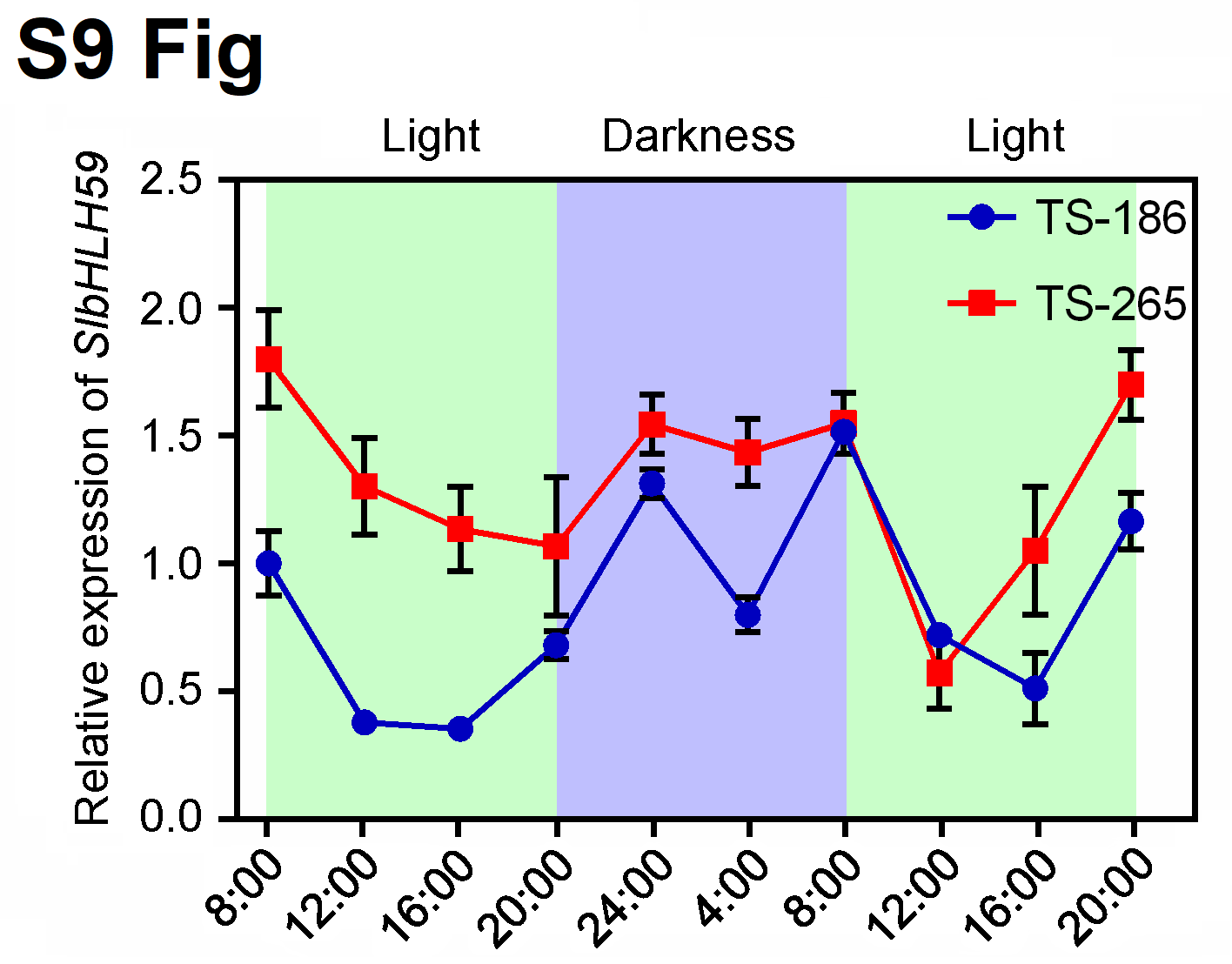

Supplement: S9 Fig — The expression of SlbHLH59 in leaves of high AsA accession TS-265 and low AsA accession TS-186 were detected. The light green indicated that the tomato plants were in the light, while the light purple indicated plants in the dark. (TIF) [file pgen.1008149.s009.tif]

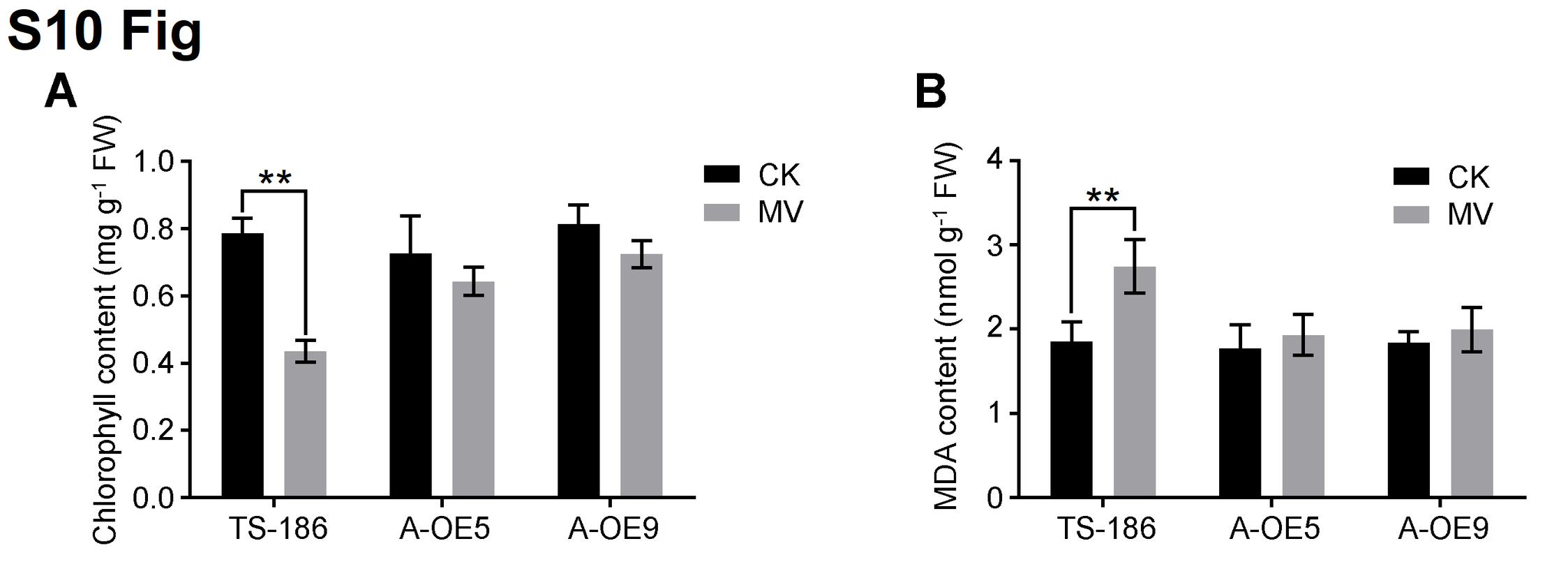

Supplement: S10 Fig — The chlorophyll (A) and malondialdehyde (MDA) (B) content in leaves were assayed 7 days after treatment with methyl viologen (MV) or water (CK). Three independent experiments were performed. The data presented are means ± SE. Asterisks represent significant differences from the control (CK), (*P < 0.05; **P < 0.01, t-test). (TIF) [file pgen.1008149.s010.tif]

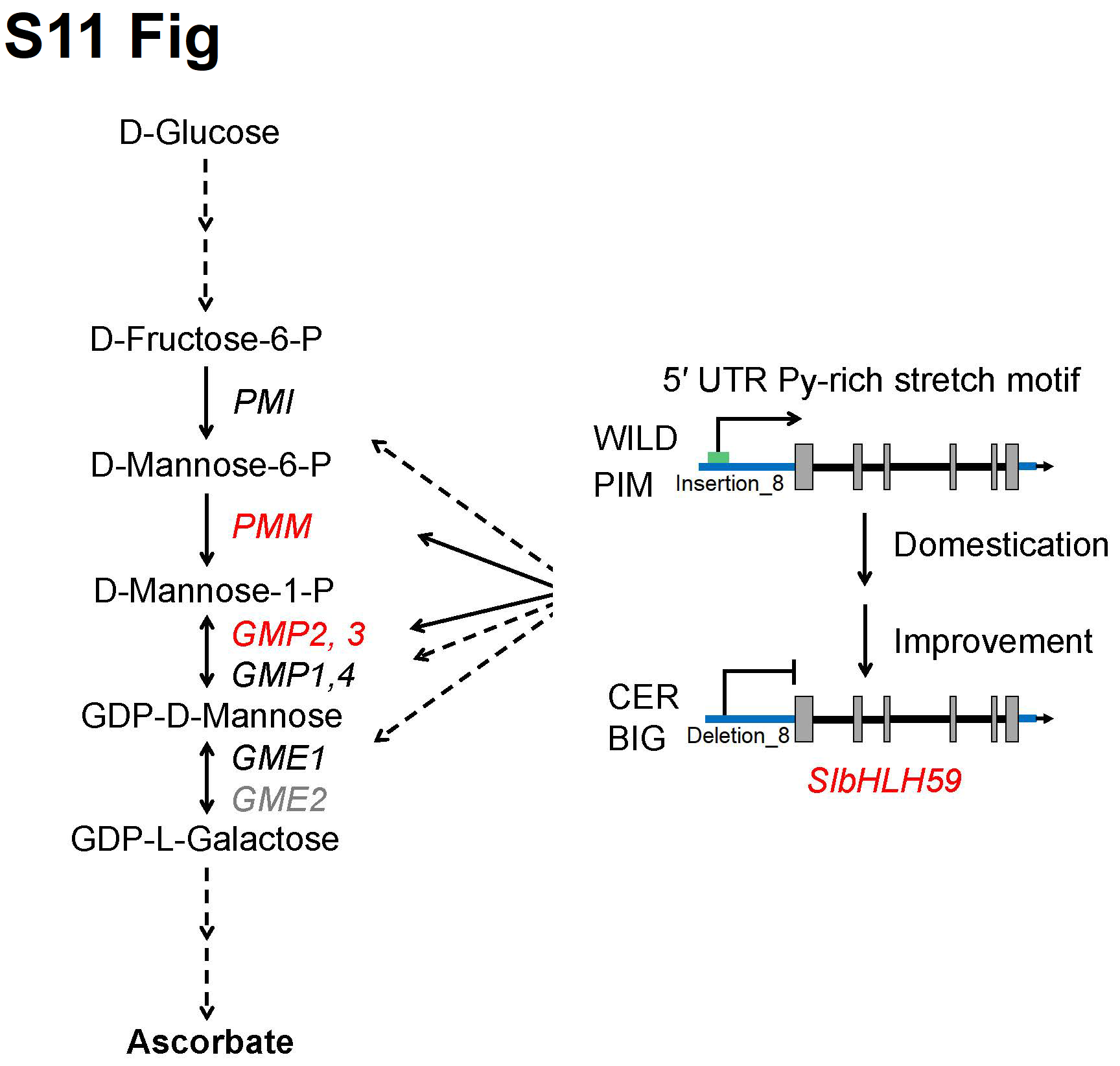

Supplement: S11 Fig — SlbHLH59 promotes the biosynthesis of AsA by positively regulating the expression of structural genes (solid arrows and red marked genes mean the directly regulation of PMM, GMP2 and GMP3; dotted arrows and black marked genes mean the indirectly regulation of PMI, GPM1, GMP4 and GME1. The InDel_8 (green box) in the promoter of SlbHLH59, occurred during the tomato domestication and improvement and causes the present/absent of 5’ UTR Py-rich stretch motif, thus affects SlbHLH59 the expression. (TIF) [file pgen.1008149.s011.tif]
