# Supplementary material for: Genome-wide association analysis identifies a natural variation in basic helix-loop-helix transcription factor regulating ascorbate biosynthesis via D-mannose/L-galactose pathway in tomato
Source: PLoS Genet. 2019 May 8;15(5):e1008149. doi: 10.1371/journal.pgen.1008149 (PMC6527244; doi:10.1371/journal.pgen.1008149)
Supplement: S1 File — (DOCX) [file pgen.1008149.s027.docx]

>Solyc01g010130.2.1

MDMGSKNDTQGQKRNKDATNFQSPNISLDWQLSGSNLTNASMGMIPNSNPLVDSVFPTIWDRPPNSSHLGFYGNNNANAQISPCIMNQHETAAIGSVPTRGSMSWNPLNSMLKGAMFVPPIPGMIPQSLAQLPADLGFIERAARFSCFSGGNFNDMMNRPLSVPESTKPCYRGPAPTWRTEEVLASSGLNSPSAVDPWKQNIRSGVDGSKDVSLPHENKTHEQSPLKIEKKNEIFARSRDEGKESVGLSGNESDEAECSGRQEEMGSAGLESSPKSLGSRKRKKYSQGTEHDRMKRVQQLPAEPDKELIETQKGDGRLHSPSSKHGGKNSKQRSQSSDPPKEDYIHVRARRGQATNSHSLAERVRREKISERMKFLQDLVPGCDKVTGKAVMLDEIINYVQSLQRQVEFLSMKLSTVNPRLDFNLDGVLTKDSQAGPSSALAFSPDNMTMTYASLHGWQSGLLQSGLPGDGNYIDAFRRSNTQLSSMSGGYRDPSSQVPSVWDDQLHNVVDMGFTSTAPLDCQDLSSLPPDQMKTEP*

>Solyc01g107140.1.1

MEPVVAMSEGEWSSLSGTCSTEEANFMAQLFGACPNEQQLPSSGLPNFWTNHESNIGGSSEVSIFSSQHHTNSSIYHFPTSTNHFQPMLLTTSMTMEHLPPTNNLIEADAVEFLNKQVNNDSIESGENIMSESVLHGKSLQLGREYDQMHQPESSKKRSQSPVDHKNKRSVKPKKNMKSSVADDEETGNNNNNNTVLHRQSSFSCCSEDESNVSSYDIYGLASSDNSKGVSLPNGKSRANRGSATDPQSLYARKRRERINERLRILQSLVPNGTKVDISTMLEEAVQYVKFLQLQIKLLSSDDLWMYSPIAYNGMDIGLDLKIGIPNPKP*

>Solyc01g109700.2.1

MNIALPEMLHNITSNGSSELSVLDRTKWQVQQQEMSYFNGQNDQLMNSFHQTAEAQQFHGLINVNDQSLNELVTRAIKPDPCMENSWGGFGTTGTNGFDYVPVGVGHGGMSHPSEMNYAISRTTSCPPTMADNVVKPKDTRLSSNRGRESFKKRKADKNQHLKEVAEEETKDKKLKECIEEEDDSSKVTTEKKSNKRSATNSSNSKENSDTSKEKSKITDDKKLDYIHVRARRGQATDSHSLAERVRREKISERMRFLQDLVPGCNKITGKAGMLDEIINYVQSLQRQVEFLSMKLAAVNPRLDIDADNFFNKDIFATSTSTFSAVGAGTSSEMLSMAQRQFNSLQQIMSSSGLEMGIVNLNEMALRRTTSAPVPIPEMFLDSSSINQVQSFQTWNTDLDNMYAMELQQGRSAQFLPHPCTGFAEAGHDLKMEM*

>Solyc02g062690.2.

MLQMSVLERQRAVLERIYNHSKQQLSSLVPQQELAHLITGCVQGNFNMFGGGDSNFVNFQEMARPSFSTISNSSITTVSPPPEKESDLSSMIAPRENVVSTKKRKAEFIEEEDCEKSPGNDSKENSKTSEVQKPDYIHVRARRGQATDSHSLAERARREKISKKMKYLQDLVPGCNKVTGKAGMLDEIINYVQSLQKQVEFLSMKLATLNPRLDLNTDNIFVKDLPSYMTTTFPPTVAVPTLSEYNMIQHQQAGSTGDVAQMLPQRRDLMSFPDTYLGSSHVTVVQPQQPTFEPDLQSLFSVGFN*

>Solyc03g005350.2.1

MADNPPEVYAADDFLEQILAIPSYASLPVTDLTAGASSENSTSGVSQLQQQPLFPLGLSLDNGFADANNTGGFQVKTEREAMNMGNLYPGLEHLQSHAVCLSVPQVHQVQPFQGHPTSSAIVTIPHQPAIRPRVRARRGQATDPHSIAERLRRERISERIKALQELAPSCNKTDRAAMLDEILDYVKFLRLQVKVLSMSRLGGTSAAAQVVADIPLQSVEGDTCESHSNQRVWEKWSDSETEQEVAKLMEEDVGTAMQYLQSKSLCIMPISLAALIYPTQQSDNQSMVKPEQAAPL*

>Solyc03g097820.1.1

MAAFSSHQLQHNNPFLLDSVFLPTSPIKMSGFFEEPNNSCIVQQFYQQEFPSNLISHENSFCLDPKSSSSISLDMDASSVTDKIESGINNNKANVSPLDKKRKSSEGSSSMTSAHSKNEKQGDNGKKKKIISKLVAKDEKKANEEAPTGYIHVRARRGQATDSHSLAERVRREKISERMKILQSLVPGCDKVTGKALMLDEIINYVQSLQNQVEFLSMKLTSLNPMYYDFGMDLDALMVRPDDQSLSGLETQMANIQQGSTTTTSQAAEVIANTNSGYQFLDNSTSLMFQQSHFPNSIPQGIGQLLWGADEQTQKIINQSGFSNNFCSFH*

>Solyc04g005130.2.1

MDPHSTIMSAFQTATNLAEIWPYHHLLDHTTNHAATKRRDDDESAIAVSTSGNALTESDSKRLKATRSNENGEYSGGNSGKSSDQPAKPPAEPPKDYIHVRARRGQATDSHSLAERARREKISDRMKILQDLVPGCNKVIGKALVLDEIINYVQSLQRQVEFLSMKLEAVNTRVTPTIEGIPTKDFGQQTFETNAMAFGSQGTREYAGGTSPDWLHMQIGGGFERTT*

>Solyc04g007300.2.1

MGDSSSSTPLDFHALNSTCNNNSSILMNSNMELLNSISQQLENDQNFSSNNIHQQHGFLSLSSNDQNFSNHHQHELNIMSNFHNDHHMNNISHDVYDPAVAVAAAQFFTLGGPSYGCTSSIPESESMLNSSNNNINIPTPHPLVSGNTTSKNTSEGRKRKRNNQKEVEKPREVVHVRAKRGQATDSHSLAERLRREKINEKLRCLQELVPGCYKTMGMAVMLDVIINYVRSLQNQIDFLSMKLSAASLFYDFNSSEMDDMDSMQGTNGYAAAQGMGKNIVGEGYGGFPQFQTSWPL*

>Solyc04g077480.2.1

MGTKENGSFNCPSTGMNRADSMPNVDPFSGSGWDPLLSLNQKGGFKGSSVVGHNEFVNLPYQSSQFVHYPSDSNLAEMVPKIPAFGNESYSELVNTFPLQEQLRGANCYANYVKNRGISTEGECQISGEGAVEVSPNGKRKISENHSLSNANKNVEGELQKAPSRDSSDCSKEQDGGKRHKTDQNVSSNLRNKQAGKQVKDDSDGGEPPKDNYVHVRAKRGQATNSHSLAERVRRERISERMRLLQELVPGCNKITGKAVMLDEIINYVQSLQQQVEFLSMKLATVNPELNFDIDRILSKEMLHQQTSNAALLGLGPGLSSSLPFPGISHGSFAGIPATTPPFHPLPQNVWDNELQSLLQMGFDSTSSMNNMGPNGRSKLDL*

>Solyc05g006650.2.1

MDPQASMMNHAGGFQSPPFNLSEIWQFPINAGEGETPYSFPLSTAAAPQNVSDDVRNNDPMVLDRRTNNYSGGGGGGAARKRNEDDESAKGVSTSGNGLTESASKRMKVTRSNENCEARGDGEGNSVKSAEQPAKPAEPPKDYIHVRARRGQATDSHSLAERARREKISERMKVLQDIVPGCNKVIGKALVLDEIINYIQSLQHQVEFLSMKLEAVNSKMPSIEGYPSKDFGQQPFDTNAMAFSSQATREYTRGTSPDWLHMQLGGGFERTT*

>Solyc05g010610.2.1

MDIEIKNDSEPEKRNDQEVSMNYQSPNVSSEWQLNGSNLTNSSMGMVDSFCPTTWDQPTTNSSNLGFCDASVQMDLGPFRAGVDSTLGPNWTPSNAVLKGGMFLPPVPMMLPQSLAQFPADSGFIERAARFSCFSGGNFGDMMNPFSIPESSMNPYYRGLSSMQGPQEVLANNGLKSPQKLQHLSNVAESSKDVSLTHRDTQRSPLKNEKKSENVAKSQDEAKEVAGVSGNESDEAGCSGRQEETEGAGEESCGKNIGSKKRKRGGQDTEPDQMKGAQQPPSEIQKGEQNLNPIASKPGGKNGKQGSQFSDPTKEEYIHVRARRGQATNSHSLAERIRREKISERMKYLQDLVPGCNKVTGKAVMLDEIINYVQSLQRQVEFLSMKLATVNPRLDFDIDGLLAKDILQSRAGPSSSLAFPPDMTMAYSPLHPPQAGLLQSGLPGYGFPSEGFRRAINPHLATTSCGPGDYKDPSSQAPNEWDNELHNIVQMGLNSSVPSSSQDLSGSLPAGQMKAEP*

>Solyc06g072520.1.1

MEANSNSFHVDSVFHVPIKMSGFFEEPNNNITSSSTLPNCVSQFYLQELSVNMSNNVHEISHNEPSHVTNKTNSSSLCSTQSKNVRDGDDGKGQKKRNGNVKREKKTKENKKKAPEEAPTGYVHVRARRGQATDSHSLAERVRREKISERMKILQALVPGCDKVTGKALMLDEIINYVQSLQNQVEFLSMKLASLNPMYYDFGMDLDALMVKPDQSWSGLEGPLLENTTSNYPHLDSSTSLMFQQLHLPNSVSQGSGHVLWSVDDQRQKMIINHSELISNNNNLSVPFH*

>Solyc06g083170.2.1

MDNDCFSNGGIQPPFHFDPKIPLNSLHSPHSDYFLNTHWDNNSTDNQYTHFDSALSSIVSSPVPSNSVNSNSSLCELIGKLGSICTSPSTPFTSNCDSTRTSCYTTPMSSPPKLHIPIMNQIGKDKVPNLGNSVVMNSPPFPSLSAAKFSCFGSRSFNGRTSQFELNNEDSRYGSGTGVMGIGNLTRISSSPCVVQNKNSSLMMCERLNLGKISGRNEECSVSEQDPNGEMGSKTRNVLNSKKRKAVKSKDFVPIVDETGKKRAKSTQGNGSNNGTVKMEEQKGNEDDGAEKETKENRKIAEPPKDYIHVRARRGQATDSHSLAERVRREKISQRMKLLQDLVPGCNKVTGKALMLDEIINYVRSLQHQVEFLSMKLASVNPRTDIHIDSLLHTEISQPSGSLHQHVFPVDGYAENLAQLPTICEDDLQSIVQMGFNQNSNQDLILQSQTFPVPNSESQMKIKM*

>Solyc07g005400.2.1

MNSLLSQQQQSQISLQDLQNGGNGGSTGGVGGLSQHSMGHSHFDPTSSHDDFLEQILSSVPSSSPWPDLSKSWDPHHHLSSPPHNPSSGEDQPPSNPFHSQFHYDDQASSLLASKLRQHQITSGGGAAAAAKALMLQQQLLLSRTLAGNGLRSPNGASGDNGLLSLPLNLSNGDQNDGVANPTNDNSVQALFNGFTGSLGQTSNQPQHFHHPQGGSMQSQSFGAPAMNQTPAASGSAGGGGGSTPAAQPKQQRVRARRGQATDPHSIAERLRRERIAERLKALQELVPNANKTDKASMLDEIIDYVKFLQLQVKVLSMSRLGGAPLVADMSSEGRGEGNVGRGGNGRASSSSNNETMTVTEHQVAKLMEEDMGSAMQYLQGKGLCLMPISLATAISTSTTRISNNPLLAPEAGGSTSPTLSALTVQSATAGKDATSLSET*

>Solyc09g057710.2.1

MEKDYFINGGIPNPALQFEPTMSFPSWNPLHSGQSLFNPNWDHSTPQFDSTLNSIVSSSPAELIGKLGTVCSSPQPILHNNSYSRPMMGKDNIPNLGSSLPPPLPADPGFAQRAAKFSCFGSRSFNGRTSPLGLNYTELSHRSAQTLGNGKLPRVSSSPSLKQGGSPLQLKNSGQTRMEMMSNNSNESVSEPSGETASKLPTELNSSSRKRKTVSRGKTKEDSPTEGVNGNRGSEADDNARAKRCKQVESNGIENGRVHMEESKDDETQKQVMEYQKPTEPPKDYIHVRARRGQATDSHSLAERVRREKISERMKLLQDLVPGCNKVTGKALMLDEIINYVQSLQRQVEFLSMKLATVNPNLDFPLSKDICQPNGSVAHPVFPIDKTSSSYQQGRSDIPNGALSQCSVDTSDNSLCRSLGMQLPPLDGFAEYLNQFPEDDLQSFVQMGFTQNPNKDMTLQSQGPHQTSHMKIEM*

>Solyc09g065820.2.1 (SlbHLH59)

MANNPSEGPSDDFFDQILGFPAYNGAEPNLAGNDAGAIPPAMMLQLNSGDGSSQFTGVGLGVGLGGGGFHGHGGGGSFPLGLSLEQGKGGFLKMDDVSAPGRRFRDDVVDSRASSSVKPGFHGQPMPSMPHPPAIRPRVRARRGQATDPHSIAERLRRERIAERIRALQELVPSVNKTDRAVMLDEIVDYIKFLRLQVKVLSMSRLGGAGAVAPLVTDIPISSVEEESSEGGNNNQPAWEKWSSDGTERQVAKLMEENVGAAMQFLQSKALCIMPISLASAIYHSQPPDTSSLVKPETNPPS*

>Solyc09g083220.2.1

MAANQPEGYADDFLEQILAIPPYSGLPVADVGTPSETTSFTSASAVSHLNSAAAAGLQQPLFPLGLSLDNGRDDVGDAGPYAVKHERDGMNIGNLYAGLEHLQSHAVRHSVPSVHHVQPFQGPPTTSTTVTVPHPPSIRPRVRARRGQATDPHSIAERLRRERISERIKALQELVPSCNKTDRAAMLDEILDYVKFLRLQVKVLSMSRLGGASAVAQLVADIPLQSVEGDSGESRSNQHIWDKWSNVDTEREVAKLMEEDVGAAMQYLQSKSLCIMPISLAALIYPTQQPDDQSLVKPEAAAPS*

>Solyc09g097870.2.1

MEKGNLFINDDNTTYEHLQNCFFNPNLDNNNSDPFESALSSMVSSPISIPNNNSGSDNFVLRELIGRLGSICNNTNNNSSSTNNSCYSTPLNSPPKLNLSMRGNLPPTQFTTDPGFAERAARFSCFATNLESNHSIKIQDVNLVQRNSEFGDSRENSSLSEQMIGQNDTNSRKRKSISKGKSSKIVNDKNESNAKRSKSEENENKVTKKEENAVLEENKDNQKATEPPKDYIHVRARRGQATDAHSLAERVRREKISERMKLLQDLVPGCNKVTGKAVMLDEIINYVQSLQRQVEFLSMKLATVNPRMDFNMEALLSKDMFQSRGSLGHNMYQSETSTQAFPYGFQSQPNQNYHKGTEFPFQINSLNPNLIRNSSMQLPPLDGFVEPTPQVPTFFEDDLNSVVQMGFGQNQNQSFPGVAGNVPNSQMKVEL*

>Solyc12g010170.1.1

MQAMNSFQSTGENGASSGEHMSHSHFDPSSSHDDFLQQILSSVPSSSPWPEISGDGHPYNFDDHQSTLLASKLRQHQINGGTSAAAAAKALMLQQQLLLSRGIAGNGGSGINGDQNDDGLNSGNDISVQALYNGFAGSLGQTSNQSQHFHHSQAQSFGAPAASLSMNQTPAASGSAGGAQPKQQKVRARRGQATDPHSIAERLRRERIAERMKSLQELVPNANKTDKASMLDEIIDYVRFLQLQVKVLSMSRLGGAAAVAPLVADRSSEGGGDCVQGNVGRGGSNGTTSSANNDSSMTMTEHQVAKLMEEDMGSAMQYLQGKGLCLMPISLATAISTSTCHSMKPNNPLLLAGGSAINGVGETGGGPSSPTLSASTVQSATMGNGGT*

>Solyc12g098620.1.1

MEKKNLFLNNVNTMNELNCTSTSFYNPNWENSSMDYQNDNILSSNSNNFGEISPNSFVGSNNNSCYTTPLNSPPRLNLSNFDHQIKGNFPNTSNNLPHFSTNLGNFSCFGGNDSQFVQNLESCKLSRNKSMKESEFGDSRENSSISQQIQLEEVGIKCQNDANSKKGKSIPKRKAKEITPKNDNVSTQNNESSSKRVKSDEKNEENQKPQDSLKDYIHVRARRGQATDAHSLAERVRREKIGERMKFLQDLVPGCNKVTGKAVMLDEIINYVQSLQCQVEFLSMKLSNLNSTTDFNAESLTSKNMFQSVGSLHHNMNSSESSVQEFPYGFQSQQGSNIQSFLTKETEFPFKINPHLDGFVEQTPQVPTFFEDHDLHSFIHMGFSQIQAQNYPGNVSTAQMKAEL*

>AT4G02590.2

MASNNPHDNLSDQTPSDDFFEQILGLPNFSASSAAGLSGVDGGLGGGAPPMMLQLGSGEEGSHMGGLGGSGPTGFHNQMFPLGLSLDQGKGPGFLRPEGGHGSGKRFSDDVVDNRCSSMKPVFHGQPMQQPPPSAPHQPTSIRPRVRARRGQATDPHSIAERLRRERIAERIRALQELVPTVNKTDRAAMIDEIVDYVKFLRLQVKVLSMSRLGGAGAVAPLVTDMPLSSSVEDETGEGGRTPQPAWEKWSNDGTERQVAKLMEENVGAAMQLLQSKALCMMPISLAMAIYHSQPPDTSSVVKPENNPPQ

>AT1G03040.1

MANNNNIPHDSISDPSPTDDFFEQILGLSNFSGSSGSGLSGIGGVGPPPMMLQLGSGNEGNHNHMGAIGGGGPVGFHNQMFPLGLSLDQGKGHGFLKPDETGKRFQDDVLDNRCSSMKPIFHGQPMSQPAPPMPHQQSTIRPRVRARRGQATDPHSIAERLRRERIAERIRSLQELVPTVNKTDRAAMIDEIVDYVKFLRLQVKVLSMSRLGGAGAVAPLVTEMPLSSSVEDETQAVWEKWSNDGTERQVAKLMEENVGAAMQLLQSKALCIMPISLAMAIYHSQPPDTSSSIVKPEMNPPP

>AT5G58010.1

MENGNGEGKGEFINQNNDFFLDSMSMLSSLPPCWDPSLPPPPPPPQSLFHALAVDAPFPDQFHHPQESGGPTMGSQEGLQPQGTVSTTSAPVVRQKPRVRARRGQATDPHSIAERLRRERIAERMKSLQELVPNTNKTDKASMLDEIIEYVRFLQLQVKVLSMSRLGGAGSVGPRLNGLSAEAGGRLNALTAPCNGLNGNGNATGSSNESLRSTEQRVAKLMEEDMGSAMQYLQGKGLCLMPISLATAISSSTTHSRGSLFNPISSAVAAEDSNVTATAVAAPEASSTMDDVSASKA

>AT2G24260.1

MMNSSLLTPSSSSSSHIQTPSTTFDHEDFLDQIFSSAPWPSVVDDAHPLPSDGFHGHDVDSRNQPIMMMPLNDGSSVHALYNGFSVAGSLPNFQIPQGSGGGLMNQQGQTQTQTQPQASASTATGGTVAAPPQSRTKIRARRGQATDPHSIAERLRRERIAERMKALQELVPNGNKTDKASMLDEIIDYVKFLQLQVKVLSMSRLGGAASVSSQISEAGGSHGNASSAMVGGSQTAGNSNDSVTMTEHQVAKLMEEDMGSAMQYLQGKGLCLMPISLATAISTATCHSRNPLIPGAVADVGGPSPPNLSGMTIQSTSTKMGSGNGKLNGNGVTERSSSIAVKEAVSVSKA

>AT4G30980.1

MNSSSLLTPSSSPSPHLQSPATFDHDDFLHHIFSSTPWPSSVLDDTPPPTSDCAPVTGFHHHDADSRNQITMIPLSHNHPNDALFNGFSTGSLPFHLPQGSGGQTQTQSQATASATTGGATAQPQTKPKVRARRGQATDPHSIAERLRRERIAERMKSLQELVPNGNKTDKASMLDEIIDYVKFLQLQVKVLSMSRLGGAASASSQISEDAGGSHENTSSSGEAKMTEHQVAKLMEEDMGSAMQYLQGKGLCLMPISLATTISTATCPSRSPFVKDTGVPLSPNLSTTIVANGNGSSLVTVKDAPSVSKP

>OS07G0182200-01

MRALQDLVPNTNKTDRAAMLDEILDYVKFLRLQVKVLSMSRLGGAGAVAQLVADIPISVKGEASDSGSKQQIWEKWSTDGTEKQVAKLMEEDIGAAMQFLQSKALCMMPISLAMAIYDTQHSQDGHSVKPEPNTPS

>OS03G0797600-01

MAGQQPQQQGPPEDDFFDQFFSLTSSFPGAAPGGRAAGDQPFSLALSLDAAAAAEASGSGKRLGVGDDAEGGGSKADRETVQLTGLFPPVFGGGGVQPPNLRPTPPTQVFHPQQSKQGGAAVGPQPPAPRPKVRARRGQATDPHSIAERLRRERIAERMRALQELVPNTNKTDRAAMLDEILDYVKFLRLQVKVLSMSRLGGAGAVAQLVADIPLSVKGEASDSGGNQQIWEKWSTDGTERQVAKLMEEDIGAAMQFLQSKALCMMPISLAMAIYDTQQTQDGQPVKHEPNTPS

>OS06G0184000-00

MQPSSRDTVAGGGGEGTQDDFFDQMLSTLPSAWADLGGGGGGAAGKSPWEVDPAAAAAASQVFDESALLASRLRHHQIGGAGGGGGEKPVMLQLSELHRQAGGGEEDGSGAFSPLPLFTDRTNVPPREEMEGGFKSPNAAAGGEHALFNGFGVHGGSGGAGQPPFGQGGSMSGQSFGGPAASGGTAPVTSSGGGGTAPPRQQRVRARRGQATDPHSIAERLRRERIAERMKSLQELVPNANKTDKASMLDEIIDYVKFLQLQVKVLSMSRLGGAAGMAPLVASMSSEGGGGGGGGGGGGTGGGMRVTEQQVAKMMEEDMGTAMQYLQGKGLCLMPISLASAISSATSSASLLSRPSIRHAGAPPQTMLDAAGPTSPAAMSNGDDPRHAKADGGAGGTQ

>OS02G0564700-01

MGGFAYPFTPSPAWSRDAVFAGSPWAAGGVSSLADALVSYGAVDDEEAAFLGKTAASSPSTARLHEQQQLLLEAELLRHGDGLGFAAMDDDGGAAMLGALEPCAMPLTDSGGPPVICSSSSNDSSGSEHSAAMPAGGGFLVGEQQQHVPPAAYAAGGVLPSMAAGEETPQSFGFGSLFNGDLLQEATVSKYHHHQQQQQLGVVPSSQPHHLNDDIDFNTGKLMSFASGQQHVTPSIDSLQIDQKEFSSGLHHLNLSSLISGPLASFNATQSHRQPAEACGGKNGGAAPFVNLSEVLPKGNGSGSAGNGAPKPRVRARRGQATDPHSIAERLRREKISDRMKDLQELVPNSNKTNKASMLDEIIDYVKFLQLQVKVLSMSRLGAAEAVVPLLTETQTESPGFLLSPRSSSGERQAGAGAVTGGLPGDQPELLDGGAMFEQEVVKLMEDNMTTAMQYLQSKGLCLMPVALASAISAQKGTSSAAVRPEKKKNGDGDGGGDEEDVKGEFDAPRRPPVGRPKEMRSRV

>OS06G0193400-01

MDYSAGSYMWPGNSGSENYNFVDGSSESYAEEGSLPPSGYFMGAGSDRSLKITENERNPTMLANGCLPYNTQAHPLSGQILPKGELPNNLLDLQQLQNSSNLRSNSIPPGVLQCNSTSGTFDAKLDTPGLAELPHALSSSIDSNGSDISAFLADVHAVSSAPTLCSAFQNVSSFMEPVNLDAFGFQGAQNVAMLNKTSLPNGNPSLFDNAAIASLHDSKEFLNGGSIPSFGTVLQALGAGGLKAAQQEQNIRNIPLPTFTSGSHLAVTDAQGPPLPSKIPPLIHDHNSEYPINHSSDVEPQANSAPGNSANAKPRTRARRGQATDPHSIAERLRREKISERMKNLQVLVPNSNKADKASMLDEIIDYVKFLQLQVKVLSMSRLGAPGAVLPLLRESQTECHSNPSLSASTISQGPPDMPDSEDSSAFEQEVVKLMETSIISAMQYLQNKGLCLMPIALASAISNQKGMAAAAAIPPEK

>OS02G0795800-00

MHLYLDYVTAAKHKNRRHGHLSNVPASSVLHELDREHYKSHSYMPWLALLELPASRLISCFAQTVICFVVSEAIMGADLMGFGIKTDIHLHILADRVAPAQSRVTRITVTEMTMWNFVLQTDKASMLDEIIDYVKFLQLQVKVLSMSRLGGASAVAPLVANMSSESNGNGNATSSSGNGEAANGSSNGDNNGGGTLRVTEQQVAKLMEEDMGSAMQYLQGKGLCLMPISLATAISSATSSSLLPRTGGGAGGSLHEGGNGTSPPLVNGTATGCDDAGGKQ
